# PC-PEP, a Comprehensive Daily Six-Month Home-Based Patient Empowerment Program Leads to Weight Loss in Men with Prostate Cancer: A Secondary Analysis of a Clinical Trial

**DOI:** 10.3390/curroncol31030127

**Published:** 2024-03-21

**Authors:** Wyatt MacNevin, Gabriela Ilie, Ricardo Rendon, Ross Mason, Jesse Spooner, Emily Chedrawe, Nikhilesh Patil, David Bowes, Greg Bailly, David Bell, Derek Wilke, Jeffery B. L. Zahavich, Cody MacDonald, Robert David Harold Rutledge

**Affiliations:** 1 Department of Urology, Dalhousie University, Halifax, NS B3H 4R2, Canada; 2 Department of Community Health and Epidemiology, Dalhousie University, Halifax, NS B3H 4R2, Canada; 3 Department of Radiation Oncology, Dalhousie University, Halifax, NS B3H 4R2, Canada; 4 Department of Kinesiology, Dalhousie University, Halifax, NS B3H 4R2, Canada

**Keywords:** prostate cancer, curative treatment, exercise intervention, radical prostatectomy, radiation, weight loss, Body Mass Index, physical fitness, behavioural intervention, weight management

## Abstract

**Background:** The Prostate Cancer—Patient Empowerment Program (PC-PEP) is a six-month daily home-based program shown to improve mental health and urinary function. This secondary analysis explores weight loss in male PC-PEP participants. **Methods:** In a randomized clinical trial with 128 men undergoing curative prostate cancer (PC) treatment, 66 received ‘early’ PC-PEP, while 62 were assigned to the ‘late’ waitlist-control group, receiving 6 months of standard-of-care treatment followed by 6 months of PC-PEP. PC-PEP comprised 182 daily emails with video-based exercise and dietary (predominantly plant-based) education, live online events, and 30 min strength training routines (using body weight and elastic bands). Weight and height data were collected via online surveys (baseline, 6 months, and 12 months) including medical chart reviews. Adherence was tracked weekly. **Results:** No attrition or adverse events were reported. At 6 months, the early PC-PEP group experienced significant weight loss, averaging 2.7 kg (*p* < 0.001) compared to the waitlist-control group. Weight loss was noted in the late intervention group of PC-PEP, albeit less pronounced than in the early group. Early PC-PEP surgery patients lost on average 1.4 kg (SE = 0.65) from the trial’s start to surgery day. High adherence to exercise and dietary recommendations was noted. **Conclusions:** PC-PEP led to significant weight loss in men undergoing curative prostate cancer treatment compared to standard-of-care.

## 1. Introduction

Prostate cancer is one of the most frequently diagnosed cancers in men, accounting for approximately 7% of all cancer diagnoses worldwide [1]. Despite the generally high survival rates associated with prostate cancer, men diagnosed with prostate cancer face an increased risk of concurrent medical comorbidities and premature mortality, frequently attributed to cardiovascular disease, diabetes, and conditions associated with being overweight [2,3,4,5]. In men with prostate cancer, obesity at diagnosis is associated with increased risks for developing prostate cancer treatment side-effects and is a risk factor for recurrence and decreased survival [6]. To further complicate this, fatigue and inactivity related to prostate cancer treatment put men at risk for weight gain and worsening long-term physical and mental health [7,8]. Weight loss holds significant importance for men undergoing curative radical prostatectomy, offering several advantages. It can lead to improved surgical outcomes, a reduction in postoperative complications, enhanced recovery, a lowered risk of disease progression, an elevated quality of life, and cardiovascular health benefits [6,7]. Additionally, weight loss may optimize the effectiveness of adjuvant treatments [6]. Behavioural weight management interventions have been proven to decrease weight and improve health outcomes in select cancer types, although comprehensive methodological assessments regarding their application in the context of prostate cancer are notably limited [9,10]. 

A recent systematic review and meta-analysis consolidating data from 79 cohort studies involving 33,910 men underscored the nuanced relationship between adiposity post-prostate cancer diagnosis and mortality outcomes. This extensive analysis revealed a J-shaped association between body mass index (BMI) and all-cause mortality and a linear increase in prostate cancer-specific mortality for BMIs over 26–27 [11]. These findings pinpoint a critical gap in current cancer care: the need for holistic management strategies that address weight and its impact on survivorship.

In response to this identified gap, the Prostate Cancer—Patient Empowerment Program (PC-PEP) was conceptualized to offer a comprehensive intervention focusing on weight management alongside mental and physical well-being improvements for post-diagnosis prostate cancer patients. The inception of PC-PEP was motivated by the potential benefits of addressing adiposity, enhancing the quality of life, and possibly extending survival for these patients.

The program’s feasibility and impact on health outcomes were initially tested in a 2019 pilot study, demonstrating promising results in participant adherence and improvements in mental and physical health over a brief period [12]. Building on this foundation, a more rigorous evaluation through a 12-month randomized clinical trial was conducted, comparing the outcomes between a PC-PEP intervention group and a wait-list control group. While significant benefits in mental distress, urinary symptoms, and sexual function were observed, the trial notably did not explore the program’s effect on weight loss [13,14].

The primary aim of our ongoing study is to conduct a secondary analysis to assess whether PC-PEP leads to significant weight loss in men with prostate cancer as compared to standard care. Additionally, we seek to understand how the timing of the intervention influences weight loss outcomes and whether different treatment modalities have distinct impacts on these results.

## 2. Materials and Methods

### 2.1. Secondary Analysis of a Randomized Clinical Trial

A secondary analysis was performed on a single institution randomized clinical trial that examined 128 men with biopsy-proven adenocarcinoma of the prostate (prostate cancer) from Nova Scotia, Canada, who participated in PC-PEP described elsewhere (see Study Protocol under Supplementary Materials) [14]. Recruitment spanned from December 2019 to January 2021, with participants referred by urologists, radiation oncologists, or self-referred through advertisements in major oncology clinics across the province. The comprehensive design of PC-PEP, a home-based program incorporating physical, mental, and social health activities through daily email and video links, is elaborated on pcpep.org and in other publications [13,14,15]. 

### 2.2. Inclusion Criteria

Participants were adult men eligible for either radical prostatectomy (specifically, robotic-assisted laparoscopic surgery) or primary/salvage radiation therapy (external beam, brachytherapy) ± hormone therapy. Key inclusion criteria required completion of primary treatment within six months post-randomization, fitness for a low-to-moderate exercise regimen, English literacy, email access, and the ability to travel to Halifax, Nova Scotia, for assessments at baseline, 6, and 12 months. The consent of all participants in the trial was obtained through an institutional Nova Scotia Health Authority (1024822)-approved protocol-specific informed consent form (ClinicalTrials.gov NCT03660085).

### 2.3. Study Design and Groups

The study randomly assigned 128 eligible participants in a 1:1 ratio to either the PC-PEP intervention group (n = 66) or a wait-list control group (n = 62). The early group received PC-PEP for the first six months of the trial, while the late group received standard care during this period and then underwent the PC-PEP intervention from months 6 to 12. Post-intervention, both groups had access to and were encouraged to continue applying the lifestyle recommendations and resources provided by PC-PEP. Participants were also invited to participate in the program’s monthly live video conferences. These conferences are an ongoing feature, intended to extend indefinitely as part of a Phase 4 Pan-Canadian and International PC-PEP Implementation Trial. This continuous support aims to facilitate long-term adherence to the beneficial lifestyle changes introduced during the intervention.

### 2.4. Exposure

The PC-PEP intervention, detailed in the study protocol [14], entailed daily emails with 3–5 min videos, produced by co-authors GI and RDHR. These videos included patient activation, educational content tailored for prostate cancer patients, covering healthy living habits, and guiding specific physical, mental, and social activities. Patients were encouraged to follow a daily exercise routine (minimum 30 min), practice pelvic floor muscle training routines three times a day for 10 min, and incorporate daily relaxation and stress reduction techniques (10 min/day) with a stress reduction biofeedback device (HeartMath^®^, Boulder Creek, CA, USA). The daily videos covered topics such as healthy eating (mostly plant-based), improving sleep quality, managing vitamin D intake, addressing intimacy and sexuality, managing erectile dysfunction, and enhancing communication skills. Additionally, participants had the option to connect weekly with two other co-participants and participate in monthly live Zoom conferences led by co-authors GI and RDHR.

The PC-PEP was individualized based on each participant’s fitness level at the start of the program, following a one-on-one session with an exercise physiotherapist. The exercise element of PC-PEP commenced with a 30 min demonstration session, either in-person or online, guided by a clinical exercise physiologist, alongside one of the study’s co-authors and oncologist (RDHR). Within this session, patients were provided concise instructions and practical training for Workouts A and B routines (Supplementary Materials) customized for each participant’s level of fitness. The workout versions (also presented on the main page of the program’s website, PCPEP.org) include Level 1 with 30 s of rest and 15 s of work, Level 2 with 20 s of rest and 20 s of work, Level 3 with 15 s of rest and 30 s of work, and Level 4 with 12 s of rest and 45 s of work. The study’s physiologist performed all the physical fitness assessments alongside the research coordinator (to ensure accuracy of measurements) and determined the optimal level of strength training for the patients’ current condition upon program entry. Additionally, the physiologist suggested appropriate timing for progressing to higher training intensities. The session also encompassed guidance on the proper utilization of the TheraBands (https://www.theraband.com, accessed on 19 March 2024) three elastic bands (red, green, and blue with 3.7-, 4.6-, and 5.8-pound resistance each, respectively, gauged at maximum stretch), each offering distinct levels of tension. Elastic resistance differs from free weights in its reliance on gravity, yet both elastic and isotonic resistance engage similar muscle systems, providing comparable strength training benefits, while avoiding the need to store cumbersome heavy weights. 

As part of the PC-PEP program, patients were instructed to engage in a minimum of 30 min of moderate to intense strength exercises on Mondays (Workout A) and Thursdays (Workout B), and a minimum of 30 min of any moderate to intense aerobic exercise that they preferred (brisk walk, skating, swimming, working in the garden, etc.) for the remaining 5 days of the week. This is aligned with recommendations for healthy adults made by the World Health Organization [16,17]. Patients were requested to view a 28 min exercise introduction video before they started the program, where the clinical exercise physiologist (JZ) and two co-authors (RDHR and GI) reviewed safety guidelines for strength and aerobic exercises. Men undergoing surgery were instructed to decrease their exercise and strength program the 3 days prior to the procedure and to slowly increase their aerobic component after the surgery once they had been cleared by their urologist or radiation oncologist that they were safe to exercise (e.g., on average about six weeks after surgery).

Throughout the program, participants received daily video messages containing dietary recommendations aligned with the 2020 Canadian Food Guide [18]. This guide promotes a balanced, predominantly plant-based diet centred on vegetables, fruits, whole grains, and protein sources like beans, mushrooms, nuts, and vegetables. It emphasizes water as the primary beverage, encourages replacing ultra-processed foods with fresh fruits and vegetables, advocates for healthy cooking methods, and recommends limiting consumption of processed foods high in sodium, sugar, and saturated fats. Additionally, the program emphasized mindful eating, considering cultural preferences in food choices, and substituting unhealthy options with healthier alternatives. PC-PEP also encouraged increased consumption of plant-based foods and reducing or eliminating red meat intake. Participants received examples of healthy meal choices and information on the benefits of whole foods and plant-based diets.

### 2.5. Physical Fitness Outcomes

Weight (in kilograms) and height (in meters) were reported during online surveys (self-reported) at baseline, 6, and 12 months. In-person assessments at baseline and 6 months were also measured by the study’s physiologist and the study’s research coordinator. These assessments included resting heart rate (beats per minute), systolic and diastolic blood pressure (mmHg), waist and hip circumference (cm), aerobic 6 min walk (m), hand grip strength (kg) measured in two trials using the CAMRY EH101 electronic dynamometer (CAMRY, Bolingbrook, IL, USA), one-minute endurance sit-to-stand chair test (count of repetitions), single-leg balance with eyes opened and closed (s), hamstring flexibility using the sit and reach test, and shoulder flexibility measuring an individual’s range of motion in the shoulder joint by assessing their ability to reach their hands behind their back and touch fingers together. The measurement of these assessments was affected by the start of the COVID pandemic (15 March 2020), and many in-person sessions had to be cancelled for the remainder of the trial due to COVID-19-related research restrictions and the implementation of public health measures to control the spread of the virus imposed by our research governing institution (NSHA). After each measurement, the two in-person raters independently reviewed and compared their recorded values to ensure consistency. This rigorous process of cross-checking after each assessment resulted in perfect agreement between the raters (Cohen’s Kappa = 1.0), indicating complete concordance in their measurements. Baseline assessments of height and weight and weight assessments at the time of surgery (radical prostatectomy) were also obtained at the end of the trial through a medical chart review by three of the co-authors and ensured 100% concordance among the raters (WM, RDHR, and CD). BMI, or Body Mass Index, used for the analysis was calculated by dividing each patient’s weight (kg) by the square of their height (m). A Taylor Digital Scale for body weight, 440 lb capacity, with a 12.2 × 13.5 inch platform was used for the assessments. In-person assessments were obtained at Dalhousie University’s gym. In-person assessments conducted during the study were meticulously compared with the measurements taken in clinical settings at the time of consultation (maximum one week prior to in-person study assessments). Additionally, these were cross-referenced with the weight and height data reported by patients through online surveys to ensure internal consistency. The high level of correlation found in these comparisons (r = 0.99 for both weight and height) validated the reliability of the data. Consequently, considering the high percentage of missing data for in-person measurements due to COVID pandemic restrictions, it was decided to use the weight, height, and the derived Body Mass Index (BMI) information obtained from the online self-reported surveys for the main study’s analyses.

### 2.6. Weekly Exercise and Dietary Recommendations Adherance

During the 6-month intervention, participants completed weekly online adherence surveys on Sundays, reporting their exercise activity and dietary intake for the previous week (Supplemenary Materials). The surveys documented the frequency (0 to 7 days/week) and intensity (0, rest, no feeling of exertion to 7, very, very hard) of completing the prescribed strength and aerobic activities, as well as the average daily duration (in minutes). Fifteen dietary questions, developed and validated by the Canadian government, assessed the intake of various food items. These questions evaluated healthy choices, such as the consumption of fruits and vegetables, and unhealthy choices, including snacks, fried foods, soda, alcohol, and red meat, in alignment with the guidelines set forth in the 2020 Canadian Food Guide [18,19].

### 2.7. Prognostic Covariates

Prognostic covariates gathered via the online survey included: patient age (in years), relationship status at the start of the trial (coded as 0 for ‘not in a relationship’ and 1 for ‘in a relationship’), Charlson Comorbidity Index at the onset of the trial, the number of days between randomization and the initiation of treatment, treatment modality (surgery coded as “1”, primary radiotherapy/salvage radiotherapy coded as “2”), and the use of medication for anxiety, depression, or both at the start of the trial [19,20,21,22,23]. These variables have been established as having a substantial impact on urologic and physical function outcomes in this population, and their inclusion in the analysis as covariates was predetermined based on prior considerations [24,25]. Treatment modality accuracy was verified at the end of the trial through medical chart reviews.

### 2.8. Sample Size Calculation

The sample size for this RCT was not based on the outcomes evaluated here, but it was determined based on the primary outcome (mental health) and was described elsewhere [14].

### 2.9. Statistical Analysis

Demographic, cancer-related, and health-related characteristics of the sample are presented in Table 1. Mean (independent *t*-tests or Mann–Whitney U tests) or count (Pearson χ^2^) comparisons of the outcomes between study arms assessed initial differences between the intervention and control groups. Group and treatment type stratified analyses for weight and BMI were assessed using two-level linear modeling (REML estimation), examining fixed effects of PC-PEP versus control over time (baseline, 6 months), while controlling for prognostic covariates. The Supplementary Materials present results in models without covariate adjustment. Exercise and dietary adherence over 26 weeks for early versus late PC-PEP groups were assessed using generalized linear mixed modeling (GLMM) with the SPSS GENLINMIXED procedure. This included a random intercept for the subject and a random slope for time, assessing the time × group (early versus late intervention) interaction with both time and group added to the model as fixed factors, incorporating REML. The distribution of the number of days the patient engaged in exercise was set to binomial with a LOGIT link. Analyses were set at 2-sided, *p* < 0.05.

Online assessments for weight at baseline, 6, and 12 months, and height at baseline had no missing data. The weekly adherence surveys had 18% missing data. The missing data breakdown for each variable by group is provided in the Supplementary Materials. Little’s MCAR test was not statistically significant (*p* = 0.6), indicating that data was missing at random. GLMM is particularly adept at handling missing data scenarios due to its ability to model data with complex random effects structures and variability across subjects. This method accommodates incomplete data by using all available information, thus providing reliable and valid results even in the presence of missing values. The missing-data rates across the physical fitness in-person measurements, however, ranged from 23% to 27% at baseline (25 to 30% for the waitlist control group and 21% for the early intervention group), and 24% to 28% at 6 months (30–34% for the waitlist control group and 18–21% for the early intervention group). The missing data breakdown for each variable by group is provided in the Supplementary Materials. Little’s MCAR test was not statistically significant (*p* = 0.4), indicating that data was missing at random. However, considering the substantial percentage of missing data and the limited sample size, a deliberate decision was made to prioritize the analysis of the weight, BMI, and adherence data based on the online surveys. We have included a two-level linear modelling (REML estimation) analysis of the intervention and waitlist control group in-person measures by time (baseline to 6 months) in the Supplementary Materials (without covariates adjustment); however, it is important to note that these analyses are presented with caution, as their interpretability is severely compromised due to the small sample size and extensive missing data, rendering them of limited meaningfulness. No missing data was observed for any of the covariates. Analyses were conducted using IBM SPSS Armonk (New York, NY, USA) statistical software version 27.0 [26].

## 3. Results

**Figure 1:**
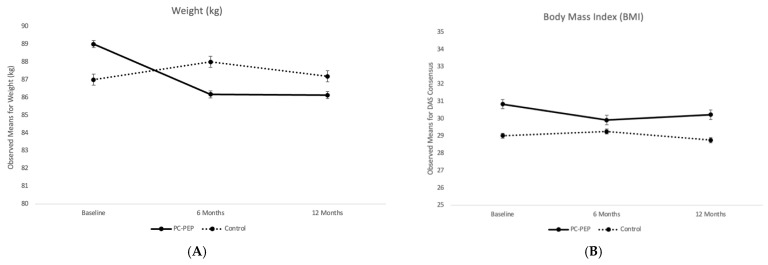
Observed means and standard errors for (**A**) weight (kilograms), and (**B**) Body Mass Index (BMI) between the waitlist-control/late PC-PEP and early PC-PEP groups at baseline, 6, and 12 months among 128 curative prostate cancer patients in the PC-PEP Phase 3 RCT, treated in Nova Scotia, Canada.

No attrition, adverse, or serious adverse events were observed during the trial. Pre-intervention characteristics for the PC-PEP and control arms were comparable (Table 1), although at the start of the trial, a higher percentage of men were in a relationship in the wait-list control group (98%) compared to the intervention group (89%). Figure 1 displays the observed means and standard errors for weight and BMI at each trial assessment time point for PC-PEP vs. waitlist-control group. 

Two-level linear modeling analyses revealed statistically significant differences between the groups (PC-PEP vs. waitlist-control) from baseline to 6 months (Table 2A) for both weight (*p* < 0.001) and BMI (*p* < 0.001). However, by the end of the year, these differences were no longer statistically significant (*p* = 0.8 for weight and *p* = 0.6 for BMI, respectively) (Table 2B). Pairwise comparisons showed a significant decrease in weight among men in the PC-PEP group at 6 months compared to baseline [−2.7 kg (95% CI: −4.05 to −1.4), *p* < 0.001], while no significant change was observed in the waitlist control group [0.68 kg (95% CI: −0.73 to 2.0), *p* = 0.4]. A similar pattern was observed for BMI, with a significant reduction among men in the PC-PEP group [−0.92 (95% CI: −1.3 to −0.49), *p* < 0.001], but not in the waitlist control group [0.24 (95% CI: −0.19 to 0.67), *p* = 0.3].

Exploratory analyses indicated no statistically significant interaction between the timing of PC-PEP delivery (early vs. late) regarding weight or BMI outcomes over time (*p* > 0.05) (Table 2C). Despite this, both (early vs. late PC-PEP) groups achieved significant reductions in weight and BMI from pre- to post-intervention [−2.3 kg (95% CI: −3.9 to −0.7), *p* = 0.005, and −0.7 (95% CI: −1.2 to −0.2), *p* = 0.009, respectively] (Figure 1), demonstrating the efficacy of PC-PEP in facilitating weight management regardless of intervention timing. It is important to note, however, that the early intervention group exhibited a trend towards greater weight loss, suggesting potential benefits of earlier engagement in the program. 

Exploratory treatment type stratified analyses, aimed at discerning whether improvements from baseline to 6 months post-trial start in the PC-PEP group were influenced by treatment modality, found no evidence supporting this hypothesis. Statistically significant group × time interactions were observed for both weight and BMI in the surgery (*p* = 0.015 and *p* = 0.013, respectively) and radiation (*p* = 0.021 and *p* = 0.01, respectively) groups (Table 3A). Post hoc comparisons show reductions in weight and BMI from pre- (trial start) to post-intervention (6 months post-trial start) for both the surgery intervention group [−2.5 kg (95% CI: −4.4 to −0.49), *p* = 0.015 and −0.79 (95% CI: −1.4 to −0.14), *p* = 0.018, respectively] and the radiation intervention group [−2.9 kg (95% CI: −4.8 to −1.1), *p* = 0.002 and −1.01 (95% CI: −1.6 to −0.45), *p* < 0.001, respectively], compared to surgery [0.91 kg (95% CI: −0.91 to 2.7), *p* = 0.3 and 0.35 (95% CI: −0.26 to 0.96), *p* = 0.3] or radiation controls [0.29 kg (95% CI: −1.8 to 2.4), *p* = 0.8 and 0.12 (95% CI: −0.52 to 0.76), *p* = 0.7, respectively]. Comparisons of weight and BMI outcomes from the start to the end of the trial (12 months later) for both early and late PC-PEP groups revealed no statistically significant interactions for surgery (*p* = 0.6 and *p* = 0.6, respectively) or the radiation groups (*p* = 0.5 and *p* = 0.4, respectively) (Table 3B). This indicates that the observed group results were not dependent on the active form of treatment patients received during the trial.

While no interactions between early versus late delivery of the PC-PEP intervention from pre to post intervention for either treatment modality emerged (Table 3C), stratified post hoc analyses reveal a statistically significant greater weight loss and registered a lower BMI from pre- to post-intervention [−2.5 kg (95% CI: −4.3 to −0.711), *p* = 0.007 and −0.79 (95% CI: −1.4 to −0.23), *p* = 0.007] for the surgery groups only, irrespective of receiving the intervention early or late. In contrast, no significant differences were observed for the radiation groups. An analysis comparing the surgery patients in the PC-PEP intervention group to the surgery patients in the control group, from the start of the trial to the time of surgery, showed that the PC-PEP surgery patients experienced an average weight loss of 1.4 kg (SE = 0.65). In contrast, the control surgery patients had an average weight gain of 0.3 kg (SE = 0.48). The statistical analysis revealed a moderate effect size: t(60) = 2.06, *p* = 0.04, Cohen’s d = 0.54 (moderate effect).

**Figure 2:**
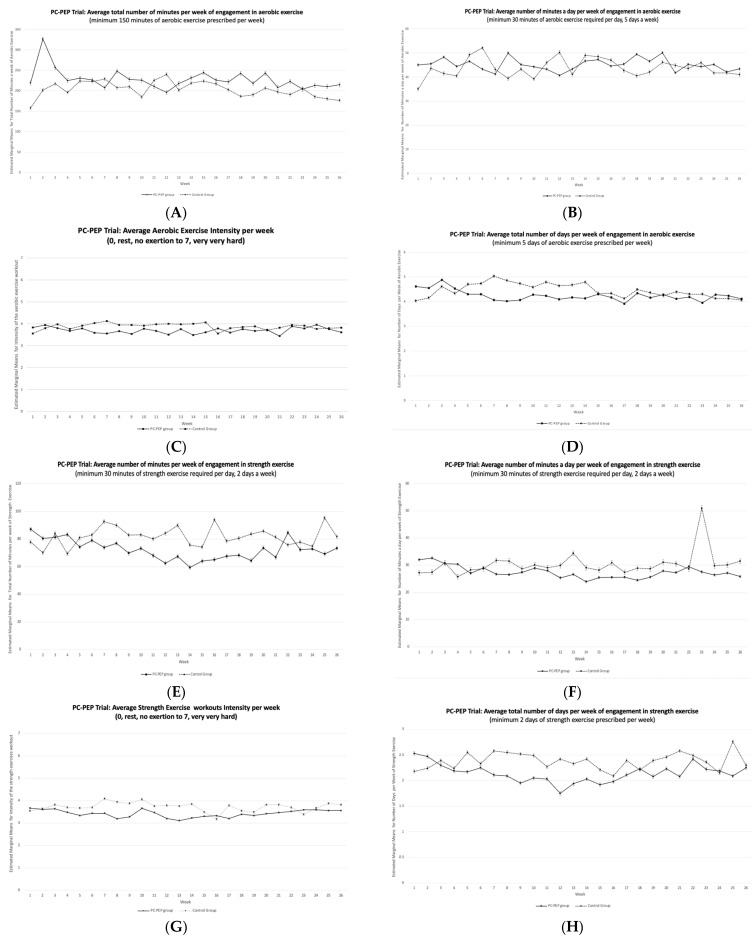
Observed means for adherence with the exercise and dietary components of the Prostate Cancer—Patient Empowerment Program (PC-PEP) program among the intervention group (who received the intervention early) and the waitlist control group (who received the intervention at 6 months post-trial start) over a 26-week period. (**A**) The total number of minutes per week of engagement in aerobic exercise, (**B**) the average number of minutes per day per week of engagement in aerobic exercise, (**C**) the number of days per week of engagement in aerobic exercise, (**D**) the average strength exercise workout intensity per week, (**E**) the average total number of minutes per week of engagement in strength exercise, (**F**) the average number of minutes per day per week of engagement in strength exercise, (**G**) the average total number of days per week of engagement in strength exercise, and (**H**) the average strength exercise workout intensity per week. These observations were made among 128 curative prostate cancer patients who underwent treatment in Nova Scotia, Canada, who participated in the PC-PEP Trial. Note: PC-PEP = Prostate Cancer—Patient Empowerment Program.

Figure 2 displays data on aerobic and strength exercise adherence over a 26-week period for both the early intervention and late intervention (waitlist-control) groups. On average, participants’ engagement in aerobic exercise ranged from 3.9 to 4.9 days per week (78% to 98%), with an overall average of 4.3 days per week (86%) for the early PC-PEP group and 4.5 days per week (90%) for the late PC-PEP group across the 26 weeks (see Supplementary Materials). In contrast, self-reported strength exercise adherence varied from 1.8 to 2.8 days per week (90% to 140%), averaging 2.1 days per week (105%) for the early PC-PEP group and 2.4 days per week (120%) for the late PC-PEP group over the course of the trial. Aerobic exercise sessions averaged 45 min daily for the early PC-PEP group and 44 min daily for the late PC-PEP group, equating to 150% and 147% adherence, respectively. Strength exercise sessions averaged 28 min daily for the early PC-PEP group and 30 min daily for the late PC-PEP group, corresponding to 93% and 100% adherence (Supplementary Materials).

Generalized linear mixed modeling (GLMM), assessing the early versus late groups over 26 weeks, revealed no significant group-by-time (26 weeks) interactions for exercise adherence (Table 4). Weekly percentages and counts of adherence to the PC-PEP’s dietary recommendations for both groups are presented in the Supplementary Materials. GLMM assessing dietary adherence over the same period showed no significant group-by-time interactions except for cigarette smoking or use of other products (*p* < 0.033) and consumption of fast-food meals or snacks (*p* < 0.030) (Table 4). Table 5 indicates that, over time, being in the early PC-PEP group acted as a protective factor against smoking cigarettes or other products (β = −0.14, t = −2.2, *p* = 0.033, OR = 0.87, 95% CI: 0.77 to 0.99) and consuming fast-food meals or snacks (β = −0.06, t = −2.2, *p* = 0.030, OR = 0.97, 95% CI: 0.89 to 0.99), compared to the late PC-PEP group.

## 4. Discussion

In this secondary analysis of a randomized controlled trial, the Prostate Cancer—Patient Empowerment Program (PC-PEP), a 6-month home-based patient empowerment program, demonstrated significant reductions in weight loss and BMI in men undergoing curative prostate cancer treatment. These benefits were observed across both early and delayed intervention timings, with marginally greater weight loss in the early intervention group. This suggests that initiating lifestyle interventions sooner may enhance their effectiveness. Surgery patients benefited from the PC-PEP program, showing weight loss prior to surgery, potentially amplified by encouragement from surgical teams. This underscores the value of preoperative weight management for optimizing surgical outcomes and postoperative recovery.

Dietary behavior changes, like decreased fast-food consumption and smoking rates observed in the early intervention group, did not alter overall dietary quality scores significantly but were associated with the greater weight loss seen in this group. This suggests that specific dietary changes, even if not affecting the overall dietary quality, contribute to weight management, emphasizing the complexity of dietary behaviors in interventions and the necessity for detailed future research on individual dietary components.

Our findings indicate that surgical patients who participated in the early phase of the PC-PEP intervention not only demonstrated weight reduction throughout the program but also significant weight loss prior to surgery. This highlights the critical role of early lifestyle interventions in facilitating preoperative weight management. The correlation between higher BMI and increased aggressiveness of tumours, as well as the heightened risk of biochemical recurrence following radical prostatectomy (RP), has been well documented [27]. Furthermore, obesity poses additional challenges during RP, particularly in surgeries utilizing robotic assistance, potentially impacting oncological outcomes by increasing the complexity of the procedure, extending operative times, and elevating the likelihood of converting to open surgery [28]. Given that over 70% of men eligible for RP fall into the overweight or obese categories, this presents a significant concern for surgical management and outcome optimization [27,28,29].

Our results may reflect not only the effectiveness of the PC-PEP but also the additional motivation provided by surgical teams, who often encourage weight loss to improve surgical outcomes and facilitate recovery. This extrinsic motivation, although not the primary focus of our investigation, appears to have complemented the PC-PEP’s influence, contributing to the substantial weight loss observed in these patients before their operations. Recognizing the combined impact of these factors is essential for a comprehensive understanding of the mechanisms underpinning the success of weight management strategies within the prostate cancer treatment framework.

Participants in the study demonstrated excellent adherence to the prescribed diet and exercise regimens, with both early and late intervention groups surpassing the recommended exercise goals. This builds on prior evidence showing mental health benefits associated with PC-PEP in men undergoing PC treatment [13,14]. Considering the association of obesity in men with PC with increased mortality risk and reduced quality of life, PC-PEP represents an important comprehensive patient empowerment strategy for men dealing with prostate cancer [14].

The necessity of focusing on quality of life improvements alongside reducing obesity-related morbidity and mortality is underscored by prostate cancer’s prolonged course and enhanced survival rates [2,5]. Obesity, known to escalate the risk of adverse treatment side-effects, reduced survival, and recurrence in prostate cancer cases, positions weight loss as a potentially key factor in positively influencing patient wellbeing [6,29,30]. While behavioural weight management interventions have been demonstrated to be effective in decreasing weight and enhancing health outcomes for several cancer types, such as breast, colon, and endometrial cancer, data regarding their efficacy in the prostate cancer patient cohort remains scarce [9,31,32,33]. Our findings advocate for the combined approach of diet and exercise over singular strategies, echoing systematic reviews that such integration yields superior weight loss and health benefits [34]. Specifically, a systematic review categorized 20 trials into three groups: diet-only (6 studies), exercise-only (8 studies), and a combination of both (6 studies), finding that while exercise-focused interventions mainly enhanced fitness and quality of life, they were less effective in reducing weight. Conversely, interventions that included dietary changes, alone or alongside exercise, observed significant weight reductions, ranging from 0.8 kg to 6.1 kg [34]. This evidence aligns with our observations in the early PC-PEP group, where surgical patients experienced an average weight loss of 1.4 kg before surgery and 2.7 kg after the 6-month intervention, underscoring the potency of lifestyle interventions that incorporate dietary modifications and structured exercise programs in facilitating weight loss among prostate cancer patients. Notably, the early PC-PEP group’s significant weight loss before and after the intervention highlights the impact of holistic lifestyle interventions in prostate cancer management.

Our study extends the existing literature by showcasing the amplified benefits of a holistic lifestyle intervention, combining dietary adjustments with increased physical activity, in a prostate cancer patient cohort. This multifaceted strategy not only led to weight loss but also enhanced overall health and well-being, emphasizing the critical role of comprehensive lifestyle interventions in cancer management. Furthermore, a separate 12-week investigation on the effects of a combined exercise and diet regimen on obese prostate cancer patients undergoing androgen deprivation therapy reported notable reductions in fat mass while preserving lean mass, along with improvements in muscle strength and cardiorespiratory fitness [8]. These results underscore the efficacy of combined lifestyle interventions, indicating that personalized exercise and dietary plans can markedly enhance health outcomes for this demographic, thereby lending further credence to our findings.

Additionally, a 2022 systematic review analyzing prostate cancer guidelines revealed a global deficiency in the emphasis on weight loss and healthy lifestyle practices among clinicians [35]. Despite the well-documented association between obesity and adverse prostate cancer outcomes, a negligible portion of these guidelines recommend weight maintenance or highlight the significance of a healthy lifestyle. This gap highlights the critical need for updating guidelines to incorporate weight management and lifestyle changes, a concern our study directly addresses through the demonstration of how a structured lifestyle intervention positively affects quality of life outcomes post prostate cancer treatment.

While many studies on weight reduction interventions in prostate cancer patients lack long-term outcomes or demonstrate high relapse rates [9], our findings indicate a stabilization of weight and BMI from 6 to 12 months among participants in the early PC-PEP group, rather than a continued reduction or significant regain. This stabilization suggests a potential for weight maintenance within this period, which we believe may be attributed to the comprehensive nature of the PC-PEP program. The initiation of the program shortly after diagnosis could play a crucial role in this outcome, as it possibly enhances patients’ motivation and commitment to maintain their health improvements. Engaging patients in their own care from the onset, particularly in the context of their daily lives, offers a contrast to the often passive reception of medical system interventions, and may be a critical factor in achieving lasting lifestyle changes [36,37].

Traditional weight reduction initiatives typically focus on aerobic exercise and dietary improvements. However, the Prostate Cancer—Patient Empowerment Program (PC-PEP) takes a more comprehensive approach, incorporating these aspects alongside mental health and social support mechanisms [14]. This holistic strategy has the potential to yield better long-term weight management results, though further longitudinal studies are necessary to substantiate these effects. Currently, a 2-year Pan-Canadian and International Trial is in progress to examine this proposition more closely (pcpep.org). Our research significantly enhances the understanding of the advantages offered by PC-PEP. It stands as the most extended intervention to date that employs daily activities and is the inaugural randomized clinical trial to assess the effects of initiating the program early (at the beginning of curative treatment) versus later (6 months post-treatment initiation) on weight loss among prostate cancer patients. The observed substantial weight loss in both the early and late PC-PEP groups demonstrates the program’s effectiveness across different phases of treatment, thereby increasing PC-PEP’s relevance and feasibility in the comprehensive care of prostate cancer patients, offering a versatile support option throughout their treatment continuum.

One of the key strengths of this study is its implementation of the Prostate Cancer—Patient Empowerment Program (PC-PEP) as a home-based initiative. Clinic-centred patient support programs can possess obstacles for patients due to inconvenience, travel issues, and financial limitations [38,39]. Through daily emails and video instruction, a home-based intervention allowed for high-quality delivery of supportive care at the patient’s choosing with increased accessibility [13,14,38,39,40,41]. This approach is particularly advantageous for reaching rural prostate cancer patients and those from lower socioeconomic backgrounds who might find clinic-based programs inaccessible.

The success of PC-PEP is also attributed to patient activation, where daily video messages encourage patients to take an active role in their healthcare [42]. This includes participating in exercise, making informed dietary choices, and seeking social support. Evidence of this activation is seen in both the early and late intervention groups, where participants exceeded the recommended weekly strength and aerobic exercise targets, reflecting their commitment to the program [13,14,42]. Importantly, the focus of the instructional content was not solely on weight loss but on fostering a lifestyle centred around healthy living. This approach underscores the program’s comprehensive nature, aiming not just for physical health improvements but for an overall enhancement of well-being through informed, health-conscious decisions.

This study encompasses several limitations warranting consideration. First, the reliance on self-reported data for monitoring exercise and dietary adherence introduces potential inaccuracies and biases, given the subjective nature of self-reporting. Furthermore, COVID-19 restrictions during the study period substantially impacted the research methodology, preventing many participants from attending in-person assessments at baseline and at 6- and 12-month intervals. Consequently, the study predominantly relied on self-reported weight measurements for the primary analysis, potentially compromising the precision of the findings.

Reflecting on these limitations, it is clear the pandemic significantly hindered our ability to accurately track and analyze longitudinal weight changes within a clinical setting. Although initial comparisons with clinical measurements validated the self-reported weight and height data, this reliance inherently limits our capacity to thoroughly observe and analyze weight changes throughout the study. This challenge highlights a vital area for future research focused on exploring the dynamics of weight change within clinical trials, especially the correlation of these changes across various study arms over time.

Despite these obstacles, the limited in-person anthropometric data collected, albeit constrained by pandemic-related restrictions, aligned with the trends observed in self-reported data (as detailed in the Supplementary Materials). While the quantity of these observations was too limited to enable meaningful analyses between study arms, they provide reassurance that self-reported data likely reflected actual weight trends within our study population accurately. Moreover, high adherence to dietary recommendations, in line with the Canadian Health Food Guide, lends further credibility to our findings. This adherence not only signifies participants’ dedication to the intervention but also bolsters the reliability of reported outcomes, effectively bridging the gap between self-reported and objective health improvement measures.

Acknowledging the necessity for a holistic view of diet quality and the risks posed by multiple comparisons, we have adopted an exploratory approach to these analyses, urging cautious interpretation of these findings. This method allows for a nuanced presentation of dietary adherence data, recognizing the methodological limitations and advocating for a consolidated approach to diet quality in subsequent research.

The strong correlation between self-reported and clinically measured baseline data suggests the feasibility of employing similar methodologies for accurately capturing longitudinal data under less restrictive conditions. Future research should, therefore, focus on developing robust tracking mechanisms for weight change in clinical settings to circumvent the challenges encountered in this study. This might include leveraging technology for enhanced self-reporting accuracy or devising protocols for safe, in-person weight measurements amid public health crises.

Additionally, examining the differential impacts of treatment arms on weight change is essential for a comprehensive understanding of treatment effects and patient outcomes. Such analyses are crucial not only for the scientific community but also for patients making informed treatment decisions. Acknowledging the limitations of our current study, we advocate for future research to prioritize these aspects, aiming for a deeper insight into the effects of treatment on patient health beyond the immediate outcomes observed in clinical trials. By recognizing these challenges and underscoring the importance of future research in this domain, we contribute to the ongoing scientific discourse on refining research methodologies in light of global health emergencies. This effort will undoubtedly expand our collective knowledge and improve our capacity to conduct significant and impactful research, even against the backdrop of societal challenges.

A nuanced limitation of this study is the absence of body fat percentage measurements, with the analysis focusing solely on weight and BMI. This omission is significant as weight loss might not fully capture the health benefits in patients who lose fat mass while gaining muscle mass, potentially underestimating the intervention’s positive health impacts. Additionally, the treatment timing for the late intervention group introduces a potential confounding variable, as these participants were not actively receiving treatment while engaging with PC-PEP materials, possibly influencing their symptomatology and energy levels differently compared to the early intervention group. Despite these factors, it is important to note that significant weight loss was observed in both intervention groups. Lastly, this study represents a secondary analysis of trial data, which may limit the findings’ scope and their applicability to broader populations or settings.

Lastly, in this study, we employed a single-item dietary screener to monitor adherence to dietary and exercise recommendations, primarily to complement our analysis of weight changes over time. While this method allowed for a streamlined integration of dietary data, we acknowledge its limitations in capturing the detailed complexity of dietary intake. The focus was on aligning with the Canadian food guide’s overall dietary recommendations, highlighting a methodological trade-off between simplicity and the depth of dietary analysis. Recognizing these constraints, there’s an opportunity for future research to utilize more detailed dietary assessment tools, enhancing the accuracy and interpretability of dietary data. Such advancements would provide a deeper insight into the intricate relationship between diet, exercise, and health outcomes, better informing public health strategies.

## 5. Conclusions

This study has shown that the Prostate Cancer—Patient Empowerment Program (PC-PEP) effectively supports men with prostate cancer in achieving healthy weight loss, demonstrating its efficacy across both early and later stages of treatment. High adherence rates to dietary and exercise guidelines underscore the program’s success, indicating that PC-PEP can significantly benefit patients without increasing the clinical workload. Moreover, PC-PEP contributes to improvements in both physical and mental health outcomes for patients, making it a valuable tool for clinicians seeking non-invasive support methods for their patients’ well-being during cancer treatment. By offering a practical approach to weight management and mental health support, PC-PEP can be easily integrated into existing clinical practices, enriching patient care with a comprehensive, patient-centred treatment component. In an era where holistic care is paramount, PC-PEP exemplifies the seamless inclusion of lifestyle interventions within oncological treatment protocols, affirming the importance of patient empowerment in enhancing recovery and overall health [8,13,14,15,42].

The adaptability of PC-PEP for clinical practice is further highlighted by its cost-effectiveness and potential to mitigate healthcare expenses. With an approximate cost of 200 CAD per patient, the program’s affordability and expanding reach across regions and cancer types make it an attractive option for widespread implementation. The ongoing implementation trial aims to assess its generalizability, catering to a broad spectrum of patients, from those on active surveillance to those with early metastatic disease.

Clinicians are tasked with screening for and managing the mental health concerns prevalent among prostate cancer patients. PC-PEP offers a clear, unburdensome pathway for achieving this goal without disrupting standard care delivery. Its early introduction post-diagnosis can prevent significant psychological distress and reduce healthcare costs, presenting a scalable solution for enhancing patient and public mental health. This model sets a new benchmark for integrating patient empowerment strategies into healthcare, promising a broader impact on patient wellness and public health.

## Figures and Tables

**Table 1:** Sample baseline characteristics comparison between the Prostate Cancer—Patient Empowerment Program (PC-PEP) intervention (n = 66) and control wait-list (n = 62) groups in a sample of prostate cancer patients undergoing curative-intent treatment in Nova Scotia, Canada (N = 128).

	PC-PEP Intervention ^a^	Wait-List Control	*p* Value
***Physical Fitness Outcomes***			
Height (meter)	66, 1.7 (0.12) [1.6, 1.9]	62, 1.7 (0.12) [1.6, 2.01]	0.103
Weight (kilogram)	66, 89 (16) [63, 141]	62, 87 (15) [68, 147]	0.6
Body Mass Index (BMI)	66, 31 (6.8) [20, 58]	62, 29 (5.7) [20, 46]	0.11
Weight (kilograms) at surgery	29, 87 (14) [70, 120]	33, 86 (13) [67, 113]	0.8
Weight difference (kilograms) between time of surgery and trial start	29, −1.4 (3.5) [−13, 4.4]	33, 0.28 (2.8) [−5.6, 6.5]	0.044
Physical activity			0.9
Not very active (<30 min/week—moderate aerobic activity)	11, 16.7%	14, 22.6%	
Moderately active (between 30–150 min/week—moderate aerobic activity)	33, 50.0%	27, 43.5%	
Very active (>150 min/week—moderate aerobic activity or 75 min/week—vigorous aerobic activity, or combination)	22, 33.3%	21, 33.9%	
***Demographic characteristics***			
Age (years)	66, 65 (7) [50, 78]	62, 67 (7) [51, 82]	0.2
Household income at baseline, >$30,000 CAD/past year	54, 82%	52, 84%	0.5
Race, White	60, 91%	61, 98%	0.063
Relationship status (currently married/in a relationship)	59, 89%	61, 98%	0.038
Education, university or greater	31, 47%	37, 60%	0.16
Employment (full or part time)	22, 33%	23, 37%	0.7
Charlson Comorbidity Index	66, 2.5 (1.1) [1, 7]	62, 2.6 (1.02) [1, 5]	0.3
***Diagnosis and treatment relevant characteristics***			
Cancer Risk Category ^b^ (RP ^c^ + primary RT ^d^ ± HT ^e^)			0.6
Low	1, 1.5%	2, 3.2%	
Intermediate	42, 75%	40, 67%	
High	13, 23%	18, 30%	
PSA (ng/mL) (salvage group only)	10, 8 (3.8) [3, 18]	2, 8 (3.3) [3, 22]	0.5
Prescribed ADT ^f^	27, 41%	21, 34%	0.4
Treatment modality			0.067
Radical prostatectomy	29, 44%	33, 53%	
Radiation therapy	27, 41%	27, 44%	
Radiation therapy ^g^ (Salvage)	10, 15%	2, 3.2%	
Time between randomization and treatment (days)	66, 61 (37) [6, 138]	62, 73 (40) [3, 173]	0.3
Use of antidepressant and/or anxiolytic for depression and/or anxiety at time of entering the trial ^h^	12, 18%	7, 11%	0.3
Absence of cancer recurrence at 6 months post-randomization	63, 96%	58, 94%	0.6

Note: Summary statistics are presented as n followed by the mean (± standard deviation) and range in square brackets, or percentage for categorical data. ^a^ Prostate Cancer—Patient Empowerment Program. ^b^ National Comprehensive Cancer Network (NCCN). ^c^ Radical prostatectomy. ^d^ Radiation therapy. ^e^ Hormone therapy. ^f^ ADT—androgen deprivation therapy. ^g^ Radiation therapy and salvage radiation groups were pooled together to allow for meaningful comparisons. ^h^ Self-identified.

**Table 2:** Two-level linear model analysis evaluating weight and body mass index (BMI) differences over time between early Prostate Cancer—Patient Empowerment Program (PC-PEP) and waitlist control groups among 128 curative prostate cancer patients in the PC-PEP randomized clinical trial, conducted in Nova Scotia, Canada.

Level	Parameter Estimate	95% Confidence Interval		*p*
		Lower	Upper	
***A. Baseline to 6 months comparison***	**Weight (kg)**			
Group (Control vs. PC-PEP)	4.3	−0.94	9.5	0.11
Time (baseline vs. 6 months)	2.7	1.4	4.05	<0.001
Time × Group (PC-PEP)	−3.4	−5.2	−1.5	<0.001
	**BMI**			
Group (Control vs. PC-PEP)	0.092	−2.2	2.3	0.9
Time (baseline vs. 6 months)	0.92	0.50	1.3	<0.001
Time × Group (PC-PEP)	−1.16	−1.8	−0.55	<0.001
***B. Baseline to 12 months comparison***	**Weight (kg)**			
Group (Control vs. early PC-PEP)	0.701	−4.6	6.0	0.8
Time (baseline vs. 12 months)	1.7	−0.83	4.3	0.19
Time × Group (PC-PEP)	−0.46	−4.1	3.2	0.8
	**BMI**			
Group (Control vs. PC-PEP)	−0.93	−3.2	1.3	0.4
Time (baseline vs. 12 months)	0.605	−0.23	1.4	0.16
Time × Group (PC-PEP)	−0.34	−1.5	0.86	0.6
***C. Early PC-PEP*** **vs. *Late PC-PEP comparison***	**Weight (kg)**			
Group (Early/baseline PC-PEP vs. Late/6-month PC-PEP)	2.1	−3.1	7.4	0.8
Time (pre vs. post-intervention)	2.7	0.51	4.9	0.016
Time × Group (Early/baseline PC-PEP)	−0.86	−4.06	2.3	0.6
	**BMI**			
Group (Early/baseline PC-PEP vs. Late/6-month PC-PEP)	−0.48	−2.8	1.9	0.6
Time (pre- vs. post-intervention)	0.92	0.17	1.65	0.015
Time × Group (Early/baseline PC-PEP)	−0.41	−1.47	0.65	0.44

Note: The following baseline covariates are included in the model: age, treatment modality, Charlson Comorbidity Index, relationship status, prescribed antidepressant/anxiolytic medication for anxiety or depression, and the number of days elapsed between trial randomization and active treatment start.

**Table 3:** Two-level linear model analysis treatment stratified evaluating weight and body mass index (BMI) outcomes over time between early PC-PEP and waitlist control groups among 128 curative prostate cancer patients in the PC-PEP randomized clinical trial, conducted in Nova Scotia, Canada.

Level	Parameter Estimate	95% Confidence Interval		*p*
		Lower	Upper	
***A. Baseline to 6 months comparison***	**Weight (kg)—Surgery**			
Group (Control vs. PC-PEP)	1.4	−4.6	7.4	0.6
Time (baseline vs. 6 months)	2.4	0.49	4.38	0.015
Time × Group (PC-PEP)	−3.3	−6.01	−0.69	0.015
	**BMI—Surgery**			
Group (Control vs. PC-PEP)	0.46	−2.4	3.4	0.8
Time (baseline vs. 6 months)	0.79	0.14	1.4	0.018
Time × Group (PC-PEP)	−1.1	−2.03	−0.25	0.013
	**Weight—Radiation**			
Group (Control vs. PC-PEP)	8.4	−0.43	17	0.062
Time (baseline vs. 6 months)	2.9	1.1	4.8	0.002
Time × Group (PC-PEP)	−3.3	−6.03	−0.50	0.021
	**BMI—Radiation**			
Group (Control vs. PC-PEP)	−0.11	−3.7	3.5	0.9
Time (baseline vs. 6 months)	1.01	0.45	1.6	<0.001
Time × Group (PC-PEP)	−1.1	−1.9	−0.28	0.01
***B. Baseline to 12 months comparison***	**Weight (kg)—Surgery**			
Group (Control vs. early PC-PEP)	−3.6	−11	3.03	0.3
Time (baseline vs. 12 months)	0.36	−2.8	3.5	0.8
Time × Group (PC-PEP)	1.3	−3.03	5.7	0.6
	**BMI—Surgery**			
Group (Control vs. PC-PEP)	−1.1	−4.2	1.9	0.5
Time (baseline vs. 12 months)	0.11	−0.94	1.2	0.8
Time × Group (PC-PEP)	0.34	−1.1	1.8	0.6
	**Weight (kg)—Radiation**			
Group (Control vs. early PC-PEP)	5.8	−2.7	14	0.18
Time (baseline vs. 12 months)	2.8	−1.2	6.7	0.17
Time × Group (PC-PEP)	−1.9	−7.9	3.9	0.5
	**BMI—Radiation**			
Group (Control vs. PC-PEP)	−0.72	−4.3	2.9	0.7
Time (baseline vs. 12 months)	0.99	−0.303	2.3	0.13
Time × Group (PC-PEP)	−0.94	−2.9	1.01	0.4
***C. Early PC-PEP*** **vs. *Late PC-PEP comparison***	**Weight (kg)—Surgery**			
Group (Early/baseline PC-PEP vs. Late/6-month PC-PEP)	−1.3	−7.8	5.2	0.7
Time (pre vs. post intervention)	2.4	−0.18	5.06	0.067
Time × Group (Early/baseline PC-PEP)	0.14	−3.5	3.7	0.9
	**BMI—Surgery**			
Group (Early/baseline PC-PEP vs. Late/6-month PC-PEP)	−0.37	−3.5	2.7	0.8
Time (pre vs. post intervention)	0.79	−0.03	1.6	0.060
Time × Group (Early/baseline PC-PEP)	0.10	−1.1	1.1	1.0
	**Weight (kg)—Radiation**			
Group (Early/baseline PC-PEP vs. Late/6-month PC-PEP)	6.5	−2.2	15	0.14
Time (pre vs. post intervention)	2.9	−0.59	6.53	0.10
Time × Group (Early/baseline PC-PEP)	−1.9	−7.3	3.5	0.5
	**BMI—Radiation**			
Group (Early/baseline PC-PEP vs. Late/6-month PC-PEP)	−0.55	−4.2	3.1	0.8
Time (pre vs. post intervention)	1.01	−0.18	2.2	0.096
Time × Group (Early/baseline PC-PEP)	−0.84	−2.6	0.97	0.4

Note: The following baseline a priori prognostic covariates are included in the model: age, treatment modality, Charlson Comorbidity Index, relationship status, prescribed antidepressant/anxiolytic medication for anxiety or depression, and the number of days elapsed between trial randomization and active treatment start.

**Table 4:** GLMM Group (late vs. early PC-PEP delivery) × Time (26 weeks) interaction evaluations of physical (aerobic and strength) and dietary recommendation, adherence among 128 prostate cancer patients from Halifax, Nova Scotia, Canada.

Tests of Fixed Effects			
Effect	*df*	*F*	*p-*Value
**Aerobic Exercises—Average number of days completed per week**			
Time	1	1.64	0.21
Group	1	0.000030	1.00
Group × Time	1	0.18	0.68
**Aerobic Exercises—Average daily number of minutes completed per week**			
Time	1	0.37	0.54
Group	1	0.59	0.45
Group × Time	1	0.60	0.44
**Aerobic Exercises—Average intensity of the workouts per week**			
Time	1	0.013	0.91
Group	1	0.32	0.57
Group × Time	1	0.00032	0.99
**Strength Exercises—Average number of days completed per week**			
Time	1	1.11	0.30
Group	1	0.16	0.69
Group × Time	1	0.61	0.44
**Strength Exercises—Average daily number of minutes completed per week**			
Time	1	0.12	0.73
Group	1	1.20	0.28
Group × Time	1	1.94	0.17
**Strength Exercises—Average intensity of the workouts per week**			
Time	1	0.62	0.43
Group	1	1.28	0.26
Group × Time	1	0.46	0.50
**Diet—Daily average of servings of fruit (1 cup) consumed**			
Time	1	1.52	0.22
Group	1	0.29	0.59
Group × Time	1	0.07	0.80
**Diet—Daily average of servings of vegetables consumed**			
Time	1	0.23	0.63
Group	1	0.12	0.73
Group × Time	1	0.01	0.93
**Diet—Daily average of servings of nuts (1 serving or 30 g) consumed**			
Time	1	11.42	0.001
Group	1	1.25	0.27
Group × Time	1	0.99	0.32
**Diet—Daily average of spoons of extra virgin olive oil consumed** **Average number per week**			
Time	1	14.47	<0.001
Group	1	0.05	0.83
Group × Time	1	0.07	0.93
**Diet—Daily average of spoons of extra virgin olive oil consumed** **Average number per week ^1^**			
Time	1	16.66	<0.001
Group	1	0.04	0.84
**Diet—Daily average of beans, chicken, white meat, or fish consumption**			
Time	1	25.09	<0.001
Group	1	2.14	0.15
Group × Time	1	1.46	0.23
**Diet—Daily average of beans, chicken, white meat, or fish consumption ^1^**			
Time	1	31.41	<0.001
Group	1	1.16	0.28
**Diet—Daily average of consumption of red meat**			
Time	2	2.69	0.073
Group	2	6.66	0.002
Group × Time	2	2.76	0.069
**Diet—Daily average of consumption of red meat ^1^**			
Time	2	2.56	0.82
Group	2	5.98	0.005
**Diet—Daily average for smoking cigarettes or other products**			
Time	1	5.38	0.022
Group	1	2.99	0.085
Group × Time	1	4.65	0.033
**Diet—Daily average of drinking wine or alcoholic beverages**			
Time	1	0.04	0.84
Group	1	7.82	0.006
Group × Time	1	3.99	0.50
**Diet—Daily average of drinking wine or alcoholic beverages ^1^**			
Time	1	0.97	0.33
Group	1	5.73	0.018
**Diet—Daily average consumption of fast-food meals or snacks**			
Time	1	1.87	0.18
Group	1	15.58	<0.001
Group × Time	1	4.86	0.030
**Diet—Daily average consumption of soda drinks**			
Time	1	0.07	0.80
Group	1	2.26	0.14
Group × Time	1	2.38	0.13
**Diet—Daily average consumption of chips or crackers**			
Time	1	0.04	0.84
Group	1	8.24	0.005
Group × Time	1	0.13	0.48
**Diet—Daily average consumption of chips or crackers ^1^**			
Time	1	0.003	0.96
Group	1	8.61	0.004
**Diet—Daily average consumption of baked goods, sweets, and pastries**			
Time	1	3.64	0.59
Group	1	1.26	0.26
Group × Time	1	2.21	0.14
**Diet—Daily average consumption of margarine, butter, and meat fat**			
Time	1	0.39	0.53
Group	1	0.52	0.47
Group × Time	1	0.034	0.86
**Diet—Daily average consumption of processed meat**			
Time	1	0.35	0.56
Group	1	6.39	0.01
Group × Time	1	0.002	0.97

^1^ Analysis displays follow-up analyses for significant main effects in the absence of a significant interaction. PC-PEP—Prostate Cancer—Patient Empowerment Program.

**Table 5:** Follow-up Prostate Cancer—Patient Empowerment Program (PC-PEP) intervention adherence analyses evaluating significant Group (late vs. early PC-PEP delivery) × Time (26 weeks) interactions, or main effects (where the interaction was found to be not significant) among 128 prostate cancer patients from Halifax, Nova Scotia, Canada.

	β	t	*p*	OR (95% CI)
**Diet—Daily average of spoons of extra virgin olive oil consumed per week**				
Time	0.072	4.08	<0.001	1.07 (1.04, 1.11)
**Diet—Daily average of beans, chicken, white meat, or fish consumption per week**				
Time	−0.061	−5.60	<0.001	0.94 (0.92, 0.96)
**Diet—Daily average of consumption of red meat per week per week**				
Group				
None (reference)				1.00
1–2 times per day	1.32	2.3	0.002	3.8 (1.7, 8.4)
≥3 times per day	1.63	3.01	0.003	5.1 (1.8–15)
**Diet—Daily average for smoking cigarettes or other products per week**				
Time	−0.005	−0.15	0.88	0.99 (0.93, −1.06)
Time × Group	−0.14	−2.2	0.033	0.87 (0.77, 0.99)
**Diet—Daily average of drinking wine or alcoholic beverages per week**				
Group	1.47	2.4	0.018	4.37 (1.3, 15)
**Diet—Daily average consumption of fast-food meals or snacks per week**				
Time	0.011	0.63	0.53	0.99 (0.98, 1.05)
Time × Group	−0.06	−2.2	0.030	0.94 (0.89, 0.99)
**Diet—Daily average consumption of chips or crackers per week**				
Group	1.08	2.94	0.004	3.0 (1.4, 6.2)

## Data Availability

Data from this study is available to researchers through a data access process in adherence with patient privacy and protection research act (NSHA Research Ethics Board).

## Supplementary Materials

The following supporting information can be downloaded at: https://www.mdpi.com/article/10.3390/curroncol31030127/s1, Figure S1: Strength exercise Workouts A and B utilized in the 6-month PC-PEP Trial; Table S1: Weekly Compliance Survey utilized in the 6 months long PC-PEP; Table S2: Missing data count for anthropomorphic in-person measurements at baseline and 6 months; Table S3: Two-level linear model analysis comparing physical fitness outcomes (in-person anthropomorphic measurements) by time (baseline compared to 6 months assessments) between the waitlist control and the early PC-PEP Intervention groups among a sample of prostate cancer patients from Halifax, Nova Scotia; Table S4: Two-level linear model analysis evaluating weight and BMI differences over time between early PC-PEP and waitlist control groups among 128 curative prostate cancer patients in the PC-PEP randomized clinical trial, conducted in Nova Scotia, Canada (no covariates added); Table S5: Two-level linear model analysis treatment stratified evaluating weight and BMI outcomes over time between early PC-PEP and waitlist control groups among 128 curative prostate cancer patients in the PC-PEP randomized clinical trial, conducted in Nova Scotia, Canada (no covariates adjustment); Table S6: Observed means and counts for the Aerobic and Strength exercise workouts Compliance over the 26 weeks of the trial among the 128 participants in the PC-PEP Trial from Halifax, Nova Scotia.
