# Supplementary material for: PC-PEP, a Comprehensive Daily Six-Month Home-Based Patient Empowerment Program Leads to Weight Loss in Men with Prostate Cancer: A Secondary Analysis of a Clinical Trial
Source: Curr Oncol. 2024 Mar 21;31(3):1667–88. doi: 10.3390/curroncol31030127 (PMC10969418; doi:10.3390/curroncol31030127)
Supplement: Supplementary file 1 [file curroncol-31-00127-s001.zip › curroncol-2904254-supplementary.pdf]

Figure S1. Strength exercise Workouts A and B utilized in the 6-month PC-PEP Trial.

Workout A		
Shoulder Press	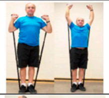	Stand or sit on the edge of a chair. Position your feet on the middle of a band. Holding onto the handles, turn your arms so your palms are facing away from you and then extend the arms and press straight overhead. Reverse the movement by lowering the arms back down in a controlled motion to the starting position. Stand/sit up tall and engage your core throughout the entire movement.
Air Squat	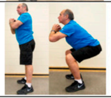	Stand with feet shoulder width apart. Bend the knees and hinge at the hips like you are sitting back into a chair until your knees reach 90 degrees. Return to the starting position and repeat. Ensure your weight is in yours and your knees are stacked directly over your ankles. Do not let the knees cave in and make sure your chest is being kept upright. Stay within a pain free range of motion.
Hydrant	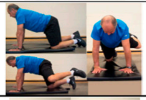	Start in a table top position; wrists directly under shoulders, knees under hips. Open one leg out to to the side (like a dog at a fire hydrant) feeling a squeeze in your glute. Return to the table top position and complete all reps before switching to the other side. The height of the leg lift is not important, your focus should be on maintaining proper form.
Chest Press	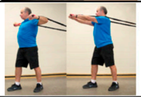	Anchor a band behind you at chest height. Stand far enough away that there is some tension on the band. Hold the band in either hand so your palms face down and extend your arms out in front. Bend your elbows to return to the starting position and repeat.
Calf Raise	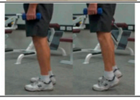	Stand with feet hip width apart on a flat surface or at the edge of a step. Lift heels to rise up onto the balls of your feet. Lower back down and repeat. To advance this exercise, try holding weights in your hands by your side or try it using one foot at a time.
Front Plank	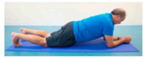	In a prone position (lying face down), position your elbows directly under your shoulders. Using either your knees or your toes as a pivot point. Ensure your body is in one straight line from the shoulders to the pivot point. Rise up into a plank and hold this position for the prescribed length of time.
Triceps Extension	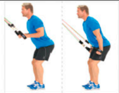	Anchor a band in front of you at chest height. Stand far enough away that there is some tension on the band. Hold the band in either hand so your palms face down and extend your arms behind you to create a contraction in muscles on the back of your upper arm. Keep your elbows tucked into your rib cage. Return to start and repeat.
Side Lunges	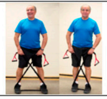	Stand on a band, cross the handles over holding one in each hand. Slightly bend your knees and lower into a squat. Keep your feet slightly internally rotated while side stepping against the band's resistance. Side step 8-12 times in one direction, and then side step back with the other foot leading.
Cat Cow	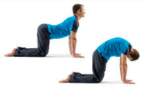	Start in a table top position; wrists directly under shoulders, knees under hips. On the inhale, swoop your low back so that your belly is pushed toward the floor, while lifting your chest and bring your gaze to the horizon (similar to a cow in the pasture). Hold for 1-2 breathes. On the exhale, reverse the pose so that your low and upper back is arched like a bridge. Draw your belly button to your spine, while tucking your chin to your chest (similar to a cat stretching in the sun).

Level 1	**15 seconds 'work' : 30 seconds 'rest'**; Each round consists of nine exercises. Perform each exercise twice before moving on to the next. Once you have completed all nine exercises, start again. If you prefer to count repetitions, **15 seconds ≈ 8-12 repetitions**. The complete workout should take approximately **27 minutes** to complete.
Level 2	**20 seconds 'work' : 20 seconds 'rest'**; Each round consists of nine exercises. Perform each exercise twice before moving on to the next. Once you have completed all nine exercises, start again. If you prefer to count repetitions, **20 seconds ≈ 12-15 repetitions**. The complete workout should take approximately **24 minutes** to complete.
Level 3	**30 seconds 'work' : 15 seconds 'rest'**; Each round consists of nine exercises. Perform each exercise twice before moving on to the next. Once you have completed all nine exercises, start again. If you prefer to count repetitions, **30 seconds ≈ 16-24 repetitions**. The complete workout should take approximately **27 minutes** to complete.
Level 4	**45 seconds 'work' : 12 seconds 'rest'**; Each round consists of nine exercises. Perform each exercise twice before moving on to the next. Once you have completed all nine exercises, start again. If you prefer to count repetitions, **45 seconds ≈ 24-36 repetitions**. The complete workout should take approximately **35 minutes** to complete.

Workout B		
Row	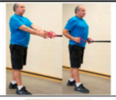	Anchor a band in front of you at chest height. Sit or stand far enough away that there is some tension on the band. Hold the band in either hand so your thumbs are pointing to the ceiling. Pull the band towards your body, elbows stay high and wide, feeling a squeeze in the muscles between your shoulder blades (upper back). Return to start and repeat.
Wall Sit	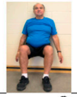	Stand with your back against a wall. Lower into a wall-supported squat. Your feet should be positioned far enough from the wall to allow for a 90 degree bend in your knees. Make sure your hips, knees and ankles maintain proper alignment.
Bird Dog	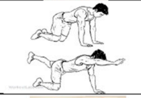	Start in a table top position; wrists directly under shoulders, knees under hips. Slowly, with control, extend opposite arm out in front, and opposite leg behind. Hold for slight pause and return to starting position. Repeat on opposite side. Throughout this exercise, maintain a neutral spine, with your head and neck aligned. Gaze on the floor.
Biceps Curl	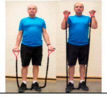	Stand with your feet shoulder width apart on a band. Hold each end of the band in your hands with palms facing up. Keeping your elbows by your side, raise the handles toward your shoulders. Slowly lower down to starting position and repeat.
6-point Lunge	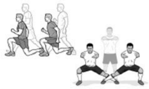	Using you left leg, step forward into a lunge. Return to standing. Reverse step into a lunge. Return to standing. Step to the side. Return to standing. Repeat this routine using your right leg.
Reverse Crunch	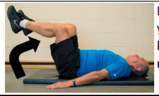	Lying flat on your back with your knees bent and feet flat on the ground. On an inhale, slowly pull your knees towards your chest, using your upper abdominals. On the exhale, slowly lower your knees until you feel your lower back arching away from the floor (end range of motion). Inhale, repeat.
Lat Pull	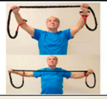	Hold a band overhead, slightly in front of you at a 45 degree angle with your hands approximately shoulder width apart. Lower the band down to the armpit level, while pulling your hands apart, so they make a rainbow shape. Reverse the movement, by raising your arms back up, while the hands return towards each other. Repeat.
Hip Lifts	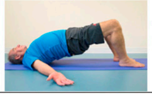	Lying flat on your back with your knees bent and feet flat on the ground. On an inhale, press through your heels and raise your hips towards the ceiling, by driving your weight into your heels. Hold for a slight pause at the top and return to your starting position. Repeat.
Windshield Wipers	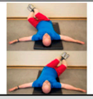	Lie flat on your back with your arms in a "T" position. Keeping your feet on the floor and knees together, with control, twist your hips so that your knees fall towards the floor. Using your lower abdominals and oblique muscles, return your knees to midline and repeat on the other side. Your head and shoulders should remain in contact with the floor. To make this exercise more challenging, try lifting your feet off the floor while maintaining a 90 degree bend in your knees.

Level 1	**15 seconds 'work' : 30 seconds 'rest'**; Each round consists of nine exercises. Perform each exercise twice before moving on to the next. Once you have completed all nine exercises, start again. If you prefer to count repetitions, **15 seconds ≈ 8-12 repetitions**. The complete workout should take approximately **27 minutes** to complete.
Level 2	**20 seconds 'work' : 20 seconds 'rest'**; Each round consists of nine exercises. Perform each exercise twice before moving on to the next. Once you have completed all nine exercises, start again. If you prefer to count repetitions, **20 seconds ≈ 12-15 repetitions**. The complete workout should take approximately **24 minutes** to complete.
Level 3	**30 seconds 'work' : 15 seconds 'rest'**; Each round consists of nine exercises. Perform each exercise twice before moving on to the next. Once you have completed all nine exercises, start again. If you prefer to count repetitions, **30 seconds ≈ 16-24 repetitions**. The complete workout should take approximately **27 minutes** to complete.
Level 4	**45 seconds 'work' : 12 seconds 'rest'**; Each round consists of nine exercises. Perform each exercise twice before moving on to the next. Once you have completed all nine exercises, start again. If you prefer to count repetitions, **45 seconds ≈ 24-36 repetitions**. The complete workout should take approximately **35 minutes** to complete.

Table S1. Compliance Survey utilized in the 6 months long PC-PEP

Compliance

Thank you for your participation in the PC-PEP (Prostate Cancer - Patient Empowerment Program)!

Please complete the survey below.

Thank you!

The following questions are about your engagement in AEROBIC EXERCISES:

During the PC-PEP Intervention/Training with our registered Certified Exercise Practitioner Jeff Zahavich, we encouraged you to do aerobic exercises (walking or substitute) for 90-150 minutes a week.

HOW MANY DAYS THIS WEEK, did you do the AEROBIC EXERCISES that you were prescribed by the Study's Exercise Expert?

- ◯ not at all
- ◯ 1 day
- ◯ 2 days
- ◯ 3 days
- ◯ 4 days
- ◯ 5 days
- ◯ 6 days
- ◯ 7 days

On average, HOW LONG (in minutes) did you spend PER DAY, during THIS WEEK, doing your AEROBIC EXERCISES?

____

For the times you did the AEROBIC EXERCISES that you were prescribed, how might you describe your exertion (how hard you worked out)?

- ◯ Rest, no feeling of exertion (e.g., this would be the equivalent of reading a book, or watching television)
- ◯ Very, very light (e.g., tying shoes)
- ◯ Very light (e.g., chores like folding clothes that seem to take little effort)
- ◯ Fairly light (e.g., walking through the grocery store or other activities that require some effort but not enough to speed up your breathing)
- ◯ Somewhat hard (e.g., brisk walking or other activities that require moderate effort and speed your heart rate and breathing but don't make you out of breath)
- ◯ Hard (e.g., bicycling, swimming, or other activities that take vigorous effort and get the heart pounding and make breathing very fast)
- ◯ Very hard (e.g., the highest level of activity you can sustain)
- ◯ Very, very hard (e.g., a finishing kick in a race or other burst of activity that you can't maintain for long)

The following questions are about your engagement in STRENGTH EXERCISES:

During the PC-PEP Intervention/Training with our registered Certified Exercise Practitioner Jeff Zahavich, we encouraged you to do strength exercises/training TWO times a week.

HOW MANY DAYS THIS WEEK, did you do the Strength EXERCISES/TRAINING that you were prescribed by the Study's Exercise Expert?

- ◯ not at all
- ◯ 1 day
- ◯ 2 days
- ◯ 3 days
- ◯ 4 days
- ◯ 5 days
- ◯ 6 days
- ◯ 7 days

On average, HOW LONG (in minutes) did you spend PER DAY, during THIS WEEK, doing your STRENGTH EXERCISES?

____

For the times you did the STRENGTH EXERCISES/TRAINING that you were prescribed, how might you describe your exertion (how hard you worked out)?

- ◯ Rest, no feeling of exertion (e.g., this would be the equivalent of reading a book, or watching television)
- ◯ Very, very light (e.g., tying shoes)
- ◯ Very light (e.g., chores like folding clothes that seem to take little effort)
- ◯ Fairly light (e.g., walking through the grocery store or other activities that require some effort but not enough to speed up your breathing)
- ◯ Somewhat hard (e.g., brisk walking or other activities that require moderate effort and speed your heart rate and breathing but don't make you out of breath)
- ◯ Hard (e.g., bicycling, swimming, or other activities that take vigorous effort and get the heart pounding and make breathing very fast)
- ◯ Very hard (e.g., the highest level of activity you can sustain)
- ◯ Very, very hard (e.g., a finishing kick in a race or other burst of activity that you can't maintain for long)

The following questions are about your engagement in PELVIC FLOOR (KEGELS) EXERCISES:

During the PC-PEP Intervention/Training with our Pelvic Floor Nurse, we encouraged you to do pelvic floor (KEGELS) exercises, 3 times a day; for 10 minutes each time.

HOW MANY DAYS THIS WEEK, did you do the PELVIC FLOOR (KEGEL) EXERCISES that you were prescribed by the Study's Physiotherapist/or Pelvic Floor Nurse?

- ◯ not at all
- ◯ 1 day
- ◯ 2 days
- ◯ 3 days
- ◯ 4 days
- ◯ 5 days
- ◯ 6 days
- ◯ 7 days

On average, HOW LONG (in minutes) did you spend PER DAY, during THIS WEEK, doing KEGELS?

____

The following questions are about your engagement in MEDITATION.

During the PC-PEP Intervention/Training, we encouraged you to meditate once a day, for 10 minutes

HOW MANY DAYS THIS WEEK, did you Meditate?

- ◯ not at all
- ◯ 1 day
- ◯ 2 days
- ◯ 3 days
- ◯ 4 days
- ◯ 5 days
- ◯ 6 days
- ◯ 7 days

On average, HOW LONG (in minutes) did you spend PER DAY, during THIS WEEK, meditating?

____

During the PC-PEP Intimacy and Connection Training of the Program we asked you to engage in at least one form of intimacy and connection every day, at least once. The following questions are about your engagement in the PC-PEP Connection and Intimacy exercises we prescribed.

HOW MANY DAYS THIS WEEK, did you engage in EMOTIONAL INTIMACY (e.g., sharing your feelings with another person, being vulnerable and authentic about how you really feel)?

- ◯ not at all
- ◯ 1 day
- ◯ 2 days
- ◯ 3 days
- ◯ 4 days
- ◯ 5 days
- ◯ 6 days
- ◯ 7 days

HOW MANY DAYS THIS WEEK, did you engage in INTELLECTUAL INTIMACY (e.g., exchanging ideas and thoughts, positive and negative, about how you think about things and how you make sense about things you care or think about)?

- ◯ not at all
- ◯ 1 day
- ◯ 2 days
- ◯ 3 days
- ◯ 4 days
- ◯ 5 days
- ◯ 6 days
- ◯ 7 days

HOW MANY DAYS THIS WEEK, did you engage in PHYSICAL INTIMACY (e.g., being in close proximity to another person/or could be a pet; touching; kissing; cuddling; caressing and/or hugging a person or could be a pet; sexual intercourse, etc.)?

- ◯ not at all
- ◯ 1 day
- ◯ 2 days
- ◯ 3 days
- ◯ 4 days
- ◯ 5 days
- ◯ 6 days
- ◯ 7 days

HOW MANY DAYS THIS WEEK, did you engage in RECREATIONAL INTIMACY (sharing in the pleasure of doing an activity together - whether active or non-active, e.g., skating, skiing, playing cards, dancing, golfing, swimming, walking, watching a movie together, hiking, traveling, doing some class together - such as a yoga class or an exercise class; or any other type of recreational activity)?

- ◯ not at all
- ◯ 1 day
- ◯ 2 days
- ◯ 3 days
- ◯ 4 days
- ◯ 5 days
- ◯ 6 days
- ◯ 7 days

HOW MANY DAYS THIS WEEK, did you engage in SELF INTIMACY (e.g., being aware of your own feelings, caring about those feelings, feeling deeply connected with how you feel and think and having love and compassion for yourself)?

- ◯ not at all
- ◯ 1 day
- ◯ 2 days
- ◯ 3 days
- ◯ 4 days
- ◯ 5 days
- ◯ 6 days
- ◯ 7 days

HOW MANY DAYS THIS WEEK, did you engage in OTHER TYPES INTIMACY/ activities where two people are mutually vulnerable, open, mindfully connected while sharing and activity. Examples may include, but not be limited to the following:

Spiritual intimacy - sharing in your spiritual journey with another, having common ethics, qualities and morals.

Unconditional intimacy- sharing in unconditionally loving someone, seeing them as the spirit that lives within rather than their appearance, their social role, their occupation or wealth.

Sleep intimacy- feeling safe and happy to share your private sleeping space with someone.

Aesthetic intimacy - sharing in the pleasure of listening to music, or watching movies, or tasking foods, or viewing works of art in an art galley.

Food intimacy - preparing and eating of the food prepared with others while connecting with one another and sharing in the pleasure of the food's taste.

Technical intimacy - sharing in reaching a common technical goal with another and working together to get there (e.g., fixing a car, a computer, and algorithm, and appliance that is not working).

Financial intimacy - figuring out together with another person how to do well with what resources you have.

- ◯ not at all
- ◯ 1 day
- ◯ 2 days
- ◯ 3 days
- ◯ 4 days
- ◯ 5 days
- ◯ 6 days
- ◯ 7 days

If you answered 1 to 7 days to the previous question please tell us what were some of the OTHER types of INTIMACY that you engaged in, during this past week? ______________________________

How many servings of fruit (1 cup) did you eat EACH DAY, on average, this week?

- ◯ None
- ◯ less than 2 servings
- ◯ 2-4 servings
- ◯ 5 or more servings on average, each day

How many servings of vegetables (fresh or cooked, not fried) did you eat EACH DAY, on average, this week? 1 serving of vegetables is 1 cup.

- ◯ None
- ◯ less than 2 servings
- ◯ 2-4 servings
- ◯ 5 or more servings on average, each day

How many servings (1 serving is the equivalent of 30 grams of mixture of peanuts, Brazilian nuts, walnuts, almonds, or hazelnuts) of nuts did you eat EACH DAY, on average, this week?

◯ I did not eat any nuts this week
◯ 2 or less
◯ 3-4 times
◯ 5 or more times on average, each day

How many spoons of extra virgin olive oil did you consume EACH DAY, on average, this week?

◯ None
◯ 3 or less
◯ 4 or more times on average, each day

How many times did you eat beans (like lentils, peas, pinto, or black beans), chicken, white - not red - meat, fish, EACH DAY, on average, this week?

◯ None
◯ less than 1 time
◯ 1-2 times
◯ 3 to 5 times
◯ more than 5 times on average, each day

How many times did you eat red meat THIS WEEK?

◯ I did not eat any red meat this week
◯ 1-2 times this week
◯ 3-4 times this week
◯ 5 times or more this week

How many times did you smoke, THIS WEEK?

◯ I did not smoke any cigarettes or other products this week
◯ Once
◯ 2-3 times
◯ 4-6 times
◯ More than 6 times

How many glasses of wine or other alcoholic beverages did you consume THIS WEEK?

◯ I did not consume any alcohol this week
◯ I only consumed wine and did so no more than 3 standard drinks a day, on average
◯ I only consumed wine and did so more than 3 standard drinks a day, on average
◯ I consumed wine AND/OR other alcoholic beverages and did so no more than 3 standard drinks a day, on average
◯ I consumed wine AND/OR other alcoholic beverages and did so more than 3 standard drinks a day, on average

How many times did you eat fast food meals or snacks EACH DAY on average, this week?

◯ I did not consume any fast food meals or snacks this week
◯ less than 1 time a day
◯ 1-2 times a day
◯ 3-4 times a day
◯ 5 or more times a day

How many regular sodas and glasses of sweet tea did you drink EACH DAY on average, this week?

◯ None
◯ 1 time or less
◯ 2-3 times
◯ 4 or more times

How many times did you eat regular snack chips or crackers (not low fat) EACH DAY on average, this week?

◯ None
◯ 1 time or less
◯ 2-3 times
◯ 4 or more times

How many times did you eat commercial bakery goods, sweets, or pastries (not low-fat kind) EACH DAY on average, this week?

◯ None
◯ 1 time or less
◯ 2-3 times
◯ 4 or more times

How much margarine, butter, or meat fat did you use to season vegetables or put on potatoes, toast, or corn EACH DAY on average, this week?

◯ None
◯ Very little (half a spoon or less)
◯ Some (between half-a-spoon and a spoon-and-a-half)
◯ A lot (more than a spoon-and-a-half)

How many times did you eat processed meats EACH DAY on average, this week?

◯ None
◯ 100 grams or less
◯ More than 100 grams each day this week on average

What dietary changes, if any, have you made in your diet THIS WEEK that you think are helpful to you in light of your prostate cancer diagnosis?

How many times did you connect with your Buddy/Buddies and/or Mentor/Mentors THIS WEEK?

◯ None
◯ Once
◯ Two or more times

Table S2: Missing data count for anthropomorphic in-person measurements at baseline and 6 months.

Physical Fitness Measures			Baseline			6-mo		
			Total	Waitlist Control	Early PCPEP	Total	Waitlist Control	Early PCPEP
Resting heart rate (bpm)	Present	Count	98	46	52	96	42	54
		Percent	76.6	74.2	78.8	75.0	67.7	81.8
	Missing	Percent	23.4	25.8	21.2	25.0	32.3	18.2
Systolic blood pressure (mmHg)	Present	Count	98	46	52	97	43	54
		Percent	76.6	74.2	78.8	75.8	69.4	81.8
	Missing	Percent	23.4	25.8	21.2	24.2	30.6	18.2
Diastolic blood pressure (mmHg)	Present	Count	98	46	52	97	43	54
		Percent	76.6	74.2	78.8	75.8	69.4	81.8
	Missing	Percent	23.4	25.8	21.2	24.2	30.6	18.2
Height (m)	Present	Count	95	44	51			
		Percent	74.2	71.0	77.3			
	Missing	Percent	25.8	29.0	22.7			
Weight (kg)	Present	Count	97	45	52	91	37	54
		Percent	75.8	72.6	78.8	71.1	59.7	81.8
	Missing	Percent	24.2	27.4	21.2	28.9	40.3	18.2
Waist circumference (cm)	Present	Count	98	46	52	97	43	54
		Percent	76.6	74.2	78.8	75.8	69.4	81.8
	Missing	Percent	23.4	25.8	21.2	24.2	30.6	18.2
Hip circumference (cm)	Present	Count	98	46	52	97	43	54
		Percent	76.6	74.2	78.8	75.8	69.4	81.8
	Missing	Percent	23.4	25.8	21.2	24.2	30.6	18.2
Aerobic 6-min walk distance (m)	Present	Count	96	44	52	92	40	52
		Percent	75.0	71.0	78.8	71.9	64.5	78.8
	Missing	Percent	25.0	29.0	21.2	28.1	35.5	21.2
Strength left hand grip (kg) -1st trial	Present	Count	98	46	52	96	42	54
		Percent	76.6	74.2	78.8	75.0	67.7	81.8

	Missing	Percent	23.4	25.8	21.2	25.0	32.3	18.2
Strength right hand grip (kg) – 1st trial	Present	Count	98	46	52	96	42	54
		Percent	76.6	74.2	78.8	75.0	67.7	81.8
	Missing	Percent	23.4	25.8	21.2	25.0	32.3	18.2
Strength left hand grip (kg) – 2nd trial	Present	Count	98	46	52	96	42	54
		Percent	76.6	74.2	78.8	75.0	67.7	81.8
	Missing	Percent	23.4	25.8	21.2	25.0	32.3	18.2
Strength right hand grip (kg) – 2nd trial	Present	Count	98	46	52	96	42	54
		Percent	76.6	74.2	78.8	75.0	67.7	81.8
	Missing	Percent	23.4	25.8	21.2	25.0	32.3	18.2
Strength hand grip combined score (kg)	Present	Count	98	46	52	96	42	54
		Percent	76.6	74.2	78.8	75.0	67.7	81.8
	Missing	Percent	23.4	25.8	21.2	25.0	32.3	18.2
Endurance sit-to-stand number of reps[1]	Present	Count	97	45	52	94	42	52
		Percent	75.8	72.6	78.8	73.4	67.7	78.8
	Missing	Percent	24.2	27.4	21.2	26.6	32.3	21.2
Balance single left leg, eyes open (s)	Present	Count	97	45	52	95	42	53
		Percent	75.8	72.6	78.8	74.2	67.7	80.3
	Missing	Percent	24.2	27.4	21.2	25.8	32.3	19.7
Balance single right leg, eyes open (s)	Present	Count	97	45	52	96	42	54
		Percent	75.8	72.6	78.8	75.0	67.7	81.8
	Missing	Percent	24.2	27.4	21.2	25.0	32.3	18.2
Balance single left leg, eyes closed (s)	Present	Count	94	42	52	94	41	53
		Percent	73.4	67.7	78.8	73.4	66.1	80.3
	Missing	Percent	26.6	32.3	21.2	26.6	33.9	19.7
Balance single right leg, eyes closed (s)	Present	Count	95	43	52	95	41	54
		Percent	74.2	69.4	78.8	74.2	66.1	81.8
	Missing	Percent	25.8	30.6	21.2	25.8	33.9	18.2
Flexibility left hamstring distance (cm)	Present	Count	97	45	52	97	43	54
		Percent	75.8	72.6	78.8	75.8	69.4	81.8

	Missing	Percent	24.2	27.4	21.2	24.2	30.6	18.2
Flexibility right hamstring distance (cm)	Present	Count	97	45	52	97	43	54
		Percent	75.8	72.6	78.8	75.8	69.4	81.8
	Missing	Percent	24.2	27.4	21.2	24.2	30.6	18.2
Flexibility left shoulder distance (cm)	Present	Count	98	46	52	97	43	54
		Percent	76.6	74.2	78.8	75.8	69.4	81.8
	Missing	Percent	23.4	25.8	21.2	24.2	30.6	18.2
Flexibility right shoulder distance (cm)	Present	Count	98	46	52	97	43	54
		Percent	76.6	74.2	78.8	75.8	69.4	81.8
	Missing	Percent	23.4	25.8	21.2	24.2	30.6	18.2

Table S3. Two-level linear model analysis comparing physical fitness outcomes (in-person anthropomorphic measurements) by time (baseline compared to 6 months assessments) between the waitlist control and the early PC-PEP Intervention groups among a sample of prostate cancer patients from Halifax, Nova Scotia.

	**Early PC-PEP**		**Wait-list Control**		***Estimate* *95% CI***	***p***
	Baseline *n, EMM (SE)*	***6-mo*** *n, EMM (SE)*	***Baseline*** *n, EMM (SE)*	***6-mo*** *n, EMM (SE)*		
Resting heart rate (bpm)	52, 69 (1.6)	54, 68 (1.6)	46, 72 (1.7)	42, 72 (1.7)	-1.5 (-5.9, 2.8)	0.5
Systolic blood pressure (mmHg)	52, 138 (2.02)	54, 136 (1.9)	46, 133 (2.1)	43, 135 (2.1)	-4.5 (-11, 2.1)	0.18
Diastolic blood pressure (mmHg)	52, 79 (1.5)	54, 79 (1.5)	46, 81 (1.6)	43, 80 (1.6)	1.5 (-3.9, 6.9)	0.6
Weight (kg)	52, 91 (2.1)	54, 88 (2.1)	46, 89 (2.2)	43, 88 (2.2)	**-2.0 (-3.6, -0.30)**	**0.022**
Body mass index	52, 29 (0.64)	54, 28 (0.65)	46, 28 (0.64)	43, 28 (0.64)	**-0.72 (-1.4, -0.10)**	**0.030**
Waist circumference (cm)	52, 104 (1.6)	54, 103 (1.6)	46, 104 (1.6)	43, 103 (1.7)	0.75 (-2.7, 1.2)	0.4
Hip circumference (cm)	52, 106 (1.1)	54, 106 (1.1)	46, 105 (1.1)	43, 105 (1.1)	-1.5 (-3.1, 0.15)	0.074
Aerobic 6-min walk distance (m)	52, 631 (13)	52, 645 (13)	44, 612 (13)	40, 604 (13)	23 (-0.41, 46)	0.054
Strength left hand grip (kg) -1st trial	52, 36 (1.1)	54, 37 (1.1)	46, 38 (1.1)	42, 37 (1.1)	1.3 (-0.72, 3.4)	0.2
Strength right hand grip (kg) – 1st trial	52, 38 (1.2) (1)	54, 38 (1.2)	46, 39 (1.1)	42, 36 (1.2)	**3.1 (0.71, 5.5)**	**0.012**
Strength left hand grip (kg) – 2nd trial	52, 38 (1.1)	54, 39 (1.1)	46, 39 (1.1)	42, 38 (1.1)	**2.9 (0.60, 5.1)**	**0.014**
Strength right hand grip (kg) – 2nd trial	52, 40 (1.1)	54, 40 (1.1)	46, 41 (1.1)	42, 39 (1.1)	1.7 (-0.15, 3.5)	0.071
Strength hand grip combined score (kg)	52, 78 (2.1)	54, 79 (2.1)	46, 81 (2.1)	42, 78 (2.1)	**4.2 (0.77, 7.6)**	**0.017**
Endurance sit-to-stand number of reps[1]	52, 17 (0.62)	52, 18 (0.62)	45, 16 (0.63)	42, 18 (0.64)	-0.47 (-1.9, 0.92)	0.5
Balance single left leg, eyes open (s)	52, 34 (2.1)	53, 32 (2.1)	45, 29 (2.2)	42, 28 (2.3)	-1.3 (-7.2, 4.6)	0.7

Balance single right leg, eyes open (s)	52, 36 (2.2)	54, 34 (2.1)	45, 32 (2.3)	42, 29 (2.3)	1.9 (-3.8, 8.8)	0.5
Balance single left leg, eyes closed (s)	52, 6.6 (0.85)	53, 3.9 (0.94)	42, 6.4 (0.93)	41, 3.9 (0.94)	0.61 (-2.7, 3.9)	0.7
Balance single right leg, eyes closed (s)	52, 5.9 (0.66)	54, 5.6 (0.66)	43, 5.0 (0.71)	41, 4.4 (0.73)	0.35 (-1.9, 2.6)	0.8
Flexibility left hamstring distance (cm)	52, -0.27 (1.5)	54, -1.3 (1.5)	45, -3.5 (1.6)	43, -2.5 (1.6)	-2.08 (-6.1, 1.9)	0.3
Flexibility right hamstring distance (cm)	52, 0.68 (1.4)	54, -0.83 (1.4)	45, -2.9 (1.5)	43, -2.5 (1.5)	-1.9 (-5.4, 1.6)	0.3
Flexibility left shoulder distance (cm)	52, -32 (2.2)	54, -33 (2.2)	46, -35 (2.3)	43, -33 (2.3)	-3.8 (-12, 4.3)	0.4
Flexibility right shoulder distance (cm)	52, -31 (1.8)	54, -29 (1.8)	46, -33 (1.9)	43, -32 (1.9)	0.71 (-3.7, 5.1)	0.8

n-group count, EMM – estimated marginal mean, SE – standard error
bpm – beats per minute; mmHg - millimeters of mercury; s – seconds; min – minutes; m – meters; cm – centimeters; kg - kilograms
[1]None of the patients required assistance.

Table S4. Two-level linear model analysis evaluating weight and BMI differences over time between early PC-PEP and waitlist control groups among 128 curative prostate cancer patients in the PC-PEP randomized clinical trial, conducted in Nova Scotia, Canada (no covariates added).

Level	*Parameter Estimate*	*95% Confidence Interval*		*p*
		Lower	*Upper*	
A. Baseline to 6 months comparison	**Weight (kg)**			
Group (Control vs PC-PEP)	1.8	-3.7	1.4	0.5
Time (baseline vs. 6 months)	2.7	1.4	4.05	<0.001
Time x Group (PC-PEP)	-3.4	-5.2	-1.5	<0.001
	**BMI**			
Group (Control vs PC-PEP)	-0.66	-2.9	1.6	0.7
Time (baseline vs. 6 months)	0.92	0.50	1.3	<0.001
Time x Group (PC-PEP)	-1.16	-1.8	-0.55	<0.001
B. Baseline to 12 months comparison	**Weight (kg)**			
Group (Control vs early PC-PEP)	-1.06	-6.5	4.4	0.7
Time (baseline vs. 12 months)	1.7	-0.83	4.3	0.19
Time x Group (PC-PEP)	-0.46	-4.1	3.2	0.8
	**BMI**			
Group (Control vs PC-PEP)	-1.5	-3.7	0.77	0.2
Time (baseline vs. 12 months)	0.61	-0.23	1.4	0.16
Time x Group (PC-PEP)	-0.34	-1.5	0.86	0.6
C. Early PC-PEP vs. Late PC-PEP comparison	**Weight (kg)**			
Group (Early/baseline PC-PEP vs. Late/6-month PC-PEP)	-0.039	-5.6	5.5	1.0
Time (pre vs post intervention)	2.7	0.51	4.9	0.016
Time x Group (Early/baseline PC-PEP)	-0.86	-4.07	2.3	0.6
	**BMI**			
Group (Early/baseline PC-PEP vs. Late/6-month PC-PEP)	-1.2	-3.5	1.1	0.3
Time (pre vs. post intervention)	0.91	0.18	1.7	0.015
Time x Group (Early/baseline PC-PEP)	-0.41	-1.5	0.65	0.4

Table S5. Two-level linear model analysis treatment stratified evaluating weight and BMI outcomes over time between early PC-PEP and waitlist control groups among 128 curative prostate cancer patients in the PC-PEP randomized clinical trial, conducted in Nova Scotia, Canada (no covariates adjustment).

Level	*Parameter Estimate*	*95% Confidence Interval*		*p*
		Lower	*Upper*	
A. Baseline to 6 months comparison	**Weight (kg) - Surgery**			
Group (Control vs PC-PEP)	0.57	-5.9	7.03	0.9
Time (baseline vs. 6 months)	2.4	0.51	4.4	0.015
Time x Group (PC-PEP)	-3.3	-6.01	-0.69	0.015
	**BMI - Surgery**			
Group (Control vs PC-PEP)	0.35	-2.6	3.3	0.8
Time (baseline vs. 6 months)	0.79	0.14	1.4	0.018
Time x Group (PC-PEP)	-1.1	-2.03	-0.25	0.013
	**Weight (kg) - Radiation**			
Group (Control vs PC-PEP)	3.4	-5.7	12	0.5
Time (baseline vs. 6 months)	2.9	1.1	4.8	0.002
Time x Group (PC-PEP)	-3.3	-6.04	-0.502	0.021
	**BMI - Radiation**			
Group (Control vs PC-PEP)	-1.4	-4.8	1.9	0.4
Time (baseline vs. 6 months)	1.01	0.4	1.6	<0.001
Time x Group (PC-PEP)	-1.1	-1.9	-0.28	0.010
B. Baseline to 12 months comparison	**Weight (kg) - Surgery**			
Group (Control vs early PC-PEP)	-4.09	-11	2.7	0.2
Time (baseline vs. 12 months)	0.36	-2.8	3.5	0.8
Time x Group (PC-PEP)	1.3	-3.03	5.7	0.6
	**BMI - Surgery**			
Group (Control vs early PC-PEP)	-1.1	-4.2	1.9	0.5
Time (baseline vs. 12 months)	0.11	-0.94	1.2	0.8
Time x Group (PC-PEP)	0.34	-1.1	1.8	0.6
	**Weight (kg) - Radiation**			
Group (Control vs early PC-PEP)	2.1	-6.5	10.7	0.6
Time (baseline vs. 12 months)	2.8	-1.2	6.7	0.17
Time x Group (PC-PEP)	-1.9	-7.9	3.9	0.5
	**BMI - Radiation**			
Group (Control vs early PC-PEP)	-1.6	-4.9	1.7	0.4
Time (baseline vs. 12 months)	0.9	-0.3	2.3	0.13
Time x Group (PC-PEP)	-0.9	-2.9	1.01	0.4
C. Early PC-PEP vs. Late PC-PEP comparison	**Weight (kg) - Surgery**			
Group (Early/baseline PC-PEP vs. Late/6-month PC-PEP)	-2.01	-8.8	4.8	0.6
Time (pre vs post intervention)	2.4	-0.18	5.06	0.067
Time x Group (Early/baseline PC-PEP)	0.14	-3.5	3.7	0.9
	**BMI - Surgery**			
Group (Early/baseline PC-PEP vs. Late/6-month PC-PEP)	-0.45	-3.5	2.6	0.8

Time (pre vs. post intervention)	0.79	-0.034	1.6	0.060
Time x Group (Early/baseline PC-PEP)	0.010	01.1	1.1	1.0
	**Weight (kg) - Radiation**			
Group (Early/baseline PC-PEP vs. Late/6-month PC-PEP)	2.3	-6.5	11	0.6
Time (pre vs post intervention)	2.9	-0.59	6.5	0.10
Time x Group (Early/baseline PC-PEP)	-1.9	-7.3	3.5	0.5
	**BMI - Radiation**			
Group (Early/baseline PC-PEP vs. Late/6-month PC-PEP)	-1.6	-5.1	1.8	0.4
Time (pre vs. post intervention)	1.01	-0.19	2.2	0.096
Time x Group (Early/baseline PC-PEP)	-0.84	-2.6	0.96	0.4

Table S6. Observed means and counts for the Aerobic and Strength exercise workouts Compliance over the 26 weeks of the trial among the 128 participants in the PC-PEP Trial from Halifax, Nova Scotia.

EARLY GROUP

Week number		1		2		3		4		5		6		7		8		9		10		11		12		13		14		15		16		17		18		19		20		21		22		23		24		25		26	
		M	n	M	n	M	n	M	n	M	n	M	n	M	n	M	n	M	n	M	n	M	n	M	n	M	n	M	n	M	n	M	n	M	n	M	n	M	n	M	n	M	n	M	n	M	n	M	n	M	n	M	n
Aerobic	Nr. of days/week	4.53	66	4.45	66	4.79	66	4.47	66	4.24	66	4.28	65	4.05	65	4.05	65	4.06	66	4.27	66	4.22	65	4.09	66	4.17	64	4.15	65	4.32	65	4.17	64	3.92	65	4.37	65	4.16	64	4.31	65	4.11	64	4.20	65	3.95	64	4.26	66	4.22	65	4.09	66
	Avg min/day	47.32	66	61.40	66	46.67	65	44.94	65	43.34	65	41.68	63	38.68	63	38.68	63	42.99	66	43.55	66	41.33	65	38.22	66	41.64	63	44.45	65	45.23	64	42.50	64	45.01	64	46.55	64	44.40	63	54.45	65	40.18	63	52.29	64	50.11	63	52.09	65	40.87	65	40.36	66
	Total min/week	222.67	66	302.71	66	251.25	65	225.05	66	219.30	66	217.97	64	200.97	64	200.97	64	225.06	66	217.76	66	205.47	65	192.73	66	216.42	63	229.27	65	244.20	64	218.24	64	224.58	64	237.95	65	219.68	63	291.60	65	208.95	63	265.67	64	251.57	64	262.46	65	215.26	65	206.89	66
	Exertion	3.82	66	3.94	66	3.80	66	3.70	66	3.77	66	3.57	65	3.57	65	3.57	65	3.56	66	3.77	66	3.66	65	3.52	66	3.75	64	3.49	65	3.62	65	3.78	64	3.60	65	3.75	65	3.67	64	3.72	65	3.44	64	3.88	65	3.78	64	3.95	66	3.75	65	3.62	66
Strength	Nr. of days/week	2.50	66	2.44	66	2.27	66	2.18	66	2.15	66	2.26	65	2.11	65	2.11	65	1.95	66	2.08	66	2.03	65	1.76	66	1.94	64	2.03	65	1.94	65	1.98	64	2.11	65	2.23	65	2.08	64	2.23	65	2.08	64	2.42	65	2.22	64	2.21	66	2.09	65	2.24	66
	Avg min/day	32.72	65	31.23	66	28.88	66	30.32	66	26.51	65	29.67	63	28.10	63	28.10	63	28.73	66	26.95	64	28.43	64	23.60	66	25.12	64	24.75	64	25.88	64	24.90	63	25.27	65	25.70	65	26.28	64	28.67	65	27.86	64	30.28	65	31.75	62	25.64	65	27.28	63	25.88	66
	Total min/week	82.37	65	77.08	66	76.48	66	78.56	66	72.39	65	75.39	64	78.33	64	78.33	64	73.22	66	69.37	64	73.37	64	55.77	66	65.14	64	64.69	64	66.92	64	64.62	63	67.87	65	70.49	65	69.66	64	79.97	65	69.41	64	85.57	65	85.44	64	73.08	66	76.08	64	70.98	66
	Exertion	3.67	66	3.62	66	3.64	66	3.50	66	3.35	66	3.45	65	3.45	65	3.45	65	3.30	66	3.67	66	3.46	65	3.23	66	3.11	64	3.25	65	3.31	65	3.33	64	3.22	65	3.40	65	3.34	64	3.43	65	3.47	64	3.52	65	3.59	64	3.61	66	3.57	65	3.58	66

LATE GROUP

Week number		1		2		3		4		5		6		7		8		9		10		11		12		13		14		15		16		17		18		19		20		21		22		23		24		25		26	
		M	n	M	n	M	n	M	n	M	n	M	n	M	n	M	n	M	n	M	n	M	n	M	n	M	n	M	n	M	n	M	n	M	n	M	n	M	n	M	n	M	n	M	n	M	n	M	n	M	n	M	n
Aerobic	Nr. of days/week	4.13	48	4.09	45	4.67	43	4.33	43	4.56	43	4.63	43	4.90	42	4.90	42	4.63	40	4.68	38	4.87	39	4.68	40	4.65	40	4.77	39	4.28	39	4.37	38	4.16	38	4.39	38	4.29	38	4.26	38	4.27	37	4.27	37	4.38	37	4.16	37	4.22	37	4.08	36
	Avg min/day	35.33	48	38.13	45	38.26	43	38.31	42	47.44	43	51.37	43	46.12	42	46.12	42	40.59	40	40.28	38	46.90	39	51.30	40	45.49	40	50.26	39	49.01	39	53.35	38	45.66	38	44.11	38	40.54	37	45.50	38	46.06	37	47.30	37	47.54	37	43.73	37	42.53	37	45.19	36
	Total min/week	168.50	48	176.87	45	200.40	43	183.70	43	215.67	43	231.16	43	241.52	42	241.52	42	196.25	40	200.84	38	241.18	39	262.44	40	235.56	40	244.33	39	241.11	39	249.55	38	228.15	38	220.23	38	183.27	37	214.16	38	218.19	37	228.59	37	233.38	37	211.81	37	200.82	37	214.40	36
	Exertion	3.46	48	3.73	45	3.93	43	3.77	43	3.91	43	4.05	43	4.10	42	4.10	42	3.90	40	4.00	38	4.00	39	3.88	40	4.00	40	3.97	39	3.51	39	3.74	38	3.79	38	3.82	38	3.71	38	3.74	38	3.86	37	3.92	37	3.73	37	3.78	37	3.78	37	3.81	36
Strength	Nr. of days/week	2.10	48	2.09	45	2.23	43	2.14	43	2.37	43	2.23	43	2.33	42	2.33	42	2.42	40	2.42	38	2.23	39	2.28	40	2.30	40	2.36	39	2.05	39	2.05	38	2.32	38	2.21	38	2.34	38	2.45	38	2.41	37	2.32	37	2.35	37	2.05	37	2.76	37	2.25	36
	Avg min/day	25.22	45	25.94	45	29.37	43	26.80	43	28.74	43	27.84	43	30.04	42	30.04	42	27.75	40	29.08	38	28.31	39	28.98	40	30.15	40	29.49	39	26.22	39	28.45	38	27.42	38	27.76	38	28.68	38	31.09	38	28.84	37	26.46	37	28.24	37	29.14	37	29.57	37	31.22	36
	Total min/week	69.76	46	67.76	45	71.28	43	63.95	43	80.40	43	76.07	43	81.86	42	81.86	42	78.88	40	78.87	38	74.31	39	77.60	40	81.08	40	79.56	39	68.33	39	89.00	38	77.21	38	76.79	38	76.58	38	83.07	38	84.16	37	72.65	37	78.38	37	74.43	37	97.65	37	80.08	36
	Exertion	3.40	48	3.53	45	3.72	43	3.84	43	3.70	43	3.79	43	3.95	42	3.95	42	3.78	40	4.05	38	3.79	39	3.73	40	3.73	40	3.82	39	3.38	39	3.08	38	3.71	38	3.61	38	3.45	38	3.79	38	3.62	37	3.57	37	3.35	37	3.59	37	3.89	37	3.78	36

Supplementary Table – Observed counts and percentages of **Dietary recommendations Compliance over the 26 weeks of the trial** among the 128 participants in the PC-PEP Trial from Halifax, Nova Scotia.

Week number	1				2				3				4			
	Early		LATE		Early		LATE		Early		LATE		Early		LATE	
	%	n	%	n	%	n	%	n	%	n	%	n	%	n	%	n
**1. Fruit**		66		48		66		45		66		43		66		43
None	3.03%	2	0.00%	0	1.52%	1	2.22%	1	1.52%	1	0.00%	0	1.52%	1	0.00%	0
less than 2 servings	25.76%	17	35.42%	17	28.79%	19	33.33%	15	27.27%	18	27.91%	12	28.79%	19	37.21%	16
2-4 servings	62.12%	41	64.58%	31	63.64%	42	60.00%	27	63.64%	42	67.44%	29	56.06%	37	60.47%	26
5 or more servings/day	9.09%	6	0.00%	0	6.06%	4	4.44%	2	7.58%	5	4.65%	2	13.64%	9	2.33%	1
**2. Vegetables**		66		48		66		45		66		43		66		43
None	0.00%	0	0.00%	0	0.00%	0	0.00%	0	0.00%	0	0.00%	0	0.00%	0	0.00%	0
less than 2 servings	18.18%	12	22.92%	11	24.24%	16	28.89%	13	27.27%	18	32.56%	14	19.70%	13	23.26%	10
2-4 servings	60.61%	40	64.58%	31	63.64%	42	60.00%	27	56.06%	37	55.81%	24	66.67%	44	67.44%	29
5 or more servings/day	21.21%	14	12.50%	6	12.12%	8	11.11%	5	16.67%	11	11.63%	5	13.64%	9	9.30%	4
**3. Nuts**		66		48		66		45		66		43		66		43
None	27.27%	18	20.83%	10	3.03%	2	15.56%	7	7.58%	5	18.60%	8	12.12%	8	9.30%	4
2 or less	36.36%	24	50.00%	24	60.61%	40	51.11%	23	45.46%	30	58.14%	25	51.52%	34	53.49%	23
3-4 times	28.79%	19	27.08%	13	27.27%	18	31.11%	14	36.36%	24	18.60%	8	28.79%	19	25.58%	11
5 or more times/day	7.58%	5	2.08%	1	9.09%	6	2.22%	1	10.61%	7	4.65%	2	7.58%	5	11.63%	5
**4. Olive oil**		66		48		66		45		66		43		66		43
None	37.88%	25	31.25%	15	30.30%	20	33.33%	15	30.30%	20	34.88%	15	27.27%	18	32.56%	14
3 or less	57.58%	38	58.33%	28	63.64%	42	57.78%	26	60.61%	40	58.14%	25	65.15%	43	58.14%	25
4 or more/day	4.55%	3	10.42%	5	6.06%	4	8.89%	4	9.09%	6	6.98%	3	7.58%	5	9.30%	4
**5. Beans, fish, and white meat**		66		48		66		45		66		43		66		43
None	6.06%	4	2.08%	1	9.09%	6	6.67%	3	4.55%	3	2.33%	1	4.55%	3	6.98%	3
less than 1 time	6.06%	4	12.50%	6	6.06%	4	8.89%	4	15.15%	10	11.63%	5	6.06%	4	13.95%	6
1-2 times	45.46%	30	47.92%	23	48.49%	32	53.33%	24	37.88%	25	46.51%	20	40.91%	27	48.84%	21
3 to 5 times	37.88%	25	29.17%	14	30.30%	20	28.89%	13	37.88%	25	34.88%	15	43.94%	29	25.58%	11
> 5 times/day	4.55%	3	8.33%	4	6.06%	4	2.22%	1	4.55%	3	4.65%	2	4.55%	3	4.65%	2
**6. Red meat**		66		48		66		45		66		43		66		43
None	39.39%	26	29.17%	14	40.91%	27	33.33%	15	48.49%	32	27.91%	12	51.52%	34	20.93%	9
1-2 times this week	53.03%	35	54.17%	26	45.46%	30	51.11%	23	45.46%	30	55.81%	24	42.42%	28	67.44%	29
3-4 times this week	6.06%	4	16.67%	8	12.12%	8	15.56%	7	6.06%	4	16.28%	7	4.55%	3	11.63%	5
5 times or more this week	1.52%	1	0.00%	0	1.52%	1	0.00%	0	0.00%	0	0.00%	0	1.52%	1	0.00%	0
**7. Smoke**		66		48		66		45		66		43		66		43
None	93.94%	62	89.58%	43	92.40%	61	93.33%	42	92.42%	61	90.70%	39	93.94%	62	95.35%	41
Once	1.52%	1	4.17%	2	0.00%	0	2.22%	1	3.03%	2	0.00%	0	1.52%	1	0.00%	0
2-3 times	1.52%	1	2.08%	1	3.00%	2	0.00%	0	1.52%	1	2.33%	1	1.52%	1	0.00%	0
4-6 times	0.00%	0	0.00%	0	0.00%	0	0.00%	0	0.00%	0	2.33%	1	0.00%	0	0.00%	0
More than 6 times	3.03%	2	4.17%	2	4.50%	3	4.44%	2	3.03%	2	4.65%	2	3.03%	2	4.65%	2
**8. Glasses of wine/alcohol**		66		48		66		45		66		43		66		43
None	37.88%	25	31.25%	15	39.39%	26	40.00%	18	43.94%	29	34.88%	15	43.94%	29	32.56%	14
Wine only; 3 or less/day on average	27.27%	18	20.83%	10	28.79%	19	13.33%	6	22.73%	15	18.60%	8	27.27%	18	20.93%	9
Wine only; more than 3/day on average	1.52%	1	8.33%	4	6.06%	4	6.67%	3	0.00%	0	2.33%	1	1.52%	1	6.98%	3
Wine AND/OR other alcohol; 3 or less/day on avg.	30.30%	20	35.42%	17	24.24%	16	40.00%	18	28.79%	19	41.86%	18	24.24%	16	37.21%	16
Wine AND/OR other alcohol; > 3/day on avg.	3.03%	2	4.17%	2	1.52%	1	0.00%	0	4.55%	3	2.33%	1	3.03%	2	2.33%	1
**9. Fast food**		66		48		66		45		66		43		66		43
None	65.15%	43	50.00%	24	66.67%	44	48.89%	22	74.24%	49	34.88%	15	68.18%	45	39.53%	17
Less than 1 time a day	30.30%	20	43.75%	21	28.79%	19	44.44%	20	24.24%	16	55.81%	24	28.79%	19	48.84%	21
1-2 times a day	4.55%	3	6.25%	3	4.55%	3	4.44%	2	1.52%	1	9.30%	4	3.03%	2	11.63%	5
3-4 times a day	0.00%	0	0.00%	0	0.00%	0	0.00%	0	0.00%	0	0.00%	0	0.00%	0	0.00%	0
5 or more times a day	0.00%	0	0.00%	0	0.00%	0	2.22%	1	0.00%	0	0.00%	0	0.00%	0	0.00%	0
**10. Soda**		66		48		66		45		66		43		66		43
None	78.79%	52	70.83%	34	77.27%	51	73.33%	33	80.30%	53	72.09%	31	86.36%	57	72.09%	31
1 time or less	15.15%	10	20.83%	10	18.18%	12	24.44%	11	18.18%	12	27.91%	12	9.09%	6	25.58%	11
2-3 times	6.06%	4	6.25%	3	4.55%	3	2.22%	1	0.00%	0	0.00%	0	1.52%	1	2.33%	1
4 or more times	0.00%	0	2.08%	1	0.00%	0	0.00%	0	1.52%	1	0.00%	0	3.03%	2	0.00%	0
**11. Chips or crackers**		66		48		66		45		66		43		66		43
None	54.55%	36	25.00%	12	54.55%	36	31.11%	14	60.61%	40	46.51%	20	59.09%	39	27.91%	12
1 times or less	37.88%	25	66.67%	32	33.33%	22	60.00%	27	28.79%	19	51.16%	22	31.82%	21	65.12%	28
2-3 times	6.06%	4	8.33%	4	12.12%	8	6.67%	3	9.09%	6	2.33%	1	7.58%	5	4.65%	2
4 or more times	1.52%	1	0.00%	0	0.00%	0	2.22%	1	1.52%	1	0.00%	0	1.52%	1	2.33%	1
**12. Baked good, sweets, and pastries**		66		48		66		45		66		43		66		43
None	46.97%	31	41.67%	20	51.52%	34	42.22%	19	51.52%	34	44.19%	19	46.97%	31	46.51%	20
1 times or less	33.33%	22	47.92%	23	33.33%	22	42.22%	19	37.88%	25	37.21%	16	39.39%	26	34.88%	15
2-3 times	16.67%	11	10.42%	5	13.64%	9	15.56%	7	10.61%	7	13.95%	6	13.64%	9	13.95%	6
4 or more times	3.03%	2	0.00%	0	1.52%	1	0.00%	0	0.00%	0	4.65%	2	0.00%	0	4.65%	2
**13. Margarine, butter, or meat fat**		66		48		66		45		66		43		66		43
None	10.61%	7	18.75%	9	16.67%	11	11.11%	5	16.67%	11	16.28%	7	25.76%	17	16.28%	7
Very little	45.46%	30	37.50%	18	39.39%	26	44.44%	20	37.88%	25	34.88%	15	31.82%	21	48.84%	21
Some	33.33%	22	35.42%	17	37.88%	25	40.00%	18	37.88%	25	39.53%	17	33.33%	22	32.56%	14
A lot	10.61%	7	8.33%	4	6.06%	4	4.44%	2	7.58%	5	9.30%	4	9.09%	6	2.33%	1
**14. Processed meats**		66		48		66		45		66		43		66		43
None	81.82%	54	58.33%	28	77.27%	51	66.67%	30	72.73%	48	62.79%	27	75.76%	50	58.14%	25
100 grams or less/day	18.18%	12	39.58%	19	22.73%	15	33.31%	15	27.27%	18	37.21%	16	24.24%	16	41.86%	18
More than 100 grams/day	0.00%	0	2.08%	1	0.00%	0	0.00%	0	0.00%	0	0.00%	0	0.00%	0	0.00%	0

Week number	5	6	7	8	9	10	11	12	13	14	15	16	17	18	19	20	21	22	23	24	25	26
Early / LATE (% , n)	[illegible]	[illegible]	[illegible]	[illegible]	[illegible]	[illegible]	[illegible]	[illegible]	[illegible]	[illegible]	[illegible]	[illegible]	[illegible]	[illegible]	[illegible]	[illegible]	[illegible]	[illegible]	[illegible]	[illegible]	[illegible]	[illegible]
